# Development and quality appraisal of a new English breast screening linked data set as part of the age, test threshold, and frequency of mammography screening (ATHENA-M) study

**DOI:** 10.1093/bjr/tqad023

**Published:** 2023-12-12

**Authors:** Julia Brettschneider, Breanna Morrison, David Jenkinson, Karoline Freeman, Jackie Walton, Alice Sitch, Sue Hudson, Olive Kearins, Alice Mansbridge, Sarah E Pinder, Rosalind Given-Wilson, Louise Wilkinson, Matthew G Wallis, Shan Cheung, Sian Taylor-Phillips

**Affiliations:** Department of Statistics, University of Warwick, Coventry, CV4 7AL, United Kingdom; University of Birmingham, Edgbaston, Birmingham, B15 2TT, United Kingdom; Warwick Medical School, University of Warwick, Coventry, CV4 7AL, United Kingdom; Warwick Medical School, University of Warwick, Coventry, CV4 7AL, United Kingdom; Screening Quality Assurance Service, NHS England, Birmingham, B2 4BH, United Kingdom; University of Birmingham, Edgbaston, Birmingham, B15 2TT, United Kingdom; Peel & Schriek Consulting Ltd, London, NW3 4QG, United Kingdom; Screening Quality Assurance Service, NHS England, Birmingham, B2 4BH, United Kingdom; Warwick Medical School, University of Warwick, Coventry, CV4 7AL, United Kingdom; School of Cancer & Pharmaceutical Sciences, King’s College London, London, WC2R 2LS, United Kingdom; Comprehensive Cancer Centre at Guy’s Hospital, Guy’s and St Thomas’ NHS Foundation Trust, London, SE1 9RT, United Kingdom; St George’s University Hospitals NHS Foundation Trust, London, SW17 0QT, United Kingdom; Oxford Breast Imaging Centre, Churchill Hospital, Oxford, OX3 7LE, United Kingdom; Cambridge Breast Unit and NIHR Cambridge Biomedical Research Centre, Cambridge University Hospitals NHS Trust, Cambridge, CB2 0QQ, United Kingdom; Screening Quality Assurance Service, NHS England, Birmingham, B2 4BH, United Kingdom; Warwick Medical School, University of Warwick, Coventry, CV4 7AL, United Kingdom

**Keywords:** breast neoplasm, mammography, data accuracy, routinely collected health data

## Abstract

**Objectives:**

To build a data set capturing the whole breast cancer screening journey from individual breast cancer screening records to outcomes and assess data quality.

**Methods:**

Routine screening records (invitation, attendance, test results) from all 79 English NHS breast screening centres between January 1, 1988 and March 31, 2018 were linked to cancer registry (cancer characteristics and treatment) and national mortality data. Data quality was assessed using comparability, validity, timeliness, and completeness.

**Results:**

Screening records were extracted from 76/79 English breast screening centres, 3/79 were not possible due to software issues. Data linkage was successful from 1997 after introduction of a universal identifier for women (NHS number). Prior to 1997 outcome data are incomplete due to linkage issues, reducing validity. Between January 1, 1997 and March 31, 2018, a total of 11 262 730 women were offered screening of whom 9 371 973 attended at least one appointment, with 139 million person-years of follow-up (a median of 12.4 person years for each woman included) with 73 810 breast cancer deaths and 1 111 139 any-cause deaths. Comparability to reference data sets and internal validity were demonstrated. Data completeness was high for core screening variables (>99%) and main cancer outcomes (>95%).

**Conclusions:**

The ATHENA-M project has created a large high-quality and representative data set of individual women’s screening trajectories and outcomes in England from 1997 to 2018, data before 1997 are lower quality.

**Advances in knowledge:**

This is the most complete data set of English breast screening records and outcomes constructed to date, which can be used to evaluate and optimize screening.

## Introduction

Data collected through screening programmes can support studies on the epidemiology of breast cancer,^1^^,^^2^ the effectiveness of screening programmes,^3^^,^^4^ the variation in cancer prevention practice due to technology or process,^5–7^ cost-effectiveness,^8–10^ potential biases,^11^ the suitability for application of AI in screening image analysis,^12^ and the potential and implementation of risk-stratification.^13^^,^^14^

Descriptions of individual breast screening observational databases in several countries have been published, including in the United States,^15–17^ Denmark,^18^ and Korea.^19^^,^^20^ However, to the best of our knowledge, to date, there is no publication reporting the data quality of routine breast screening data. Available studies focus on the quality and audit of the breast screening programme rather than the screening data itself.^21^^,^^22^ This is also true for other cancer screening.^23^^,^^24^

Three features that make English screening data sets particularly attractive are the volume of data (up to 30-year follow-up for 13 million women), inclusion of large parts of the eligible population and the relatively homogeneous organization under the umbrella of a national health system. Less systematic approaches bear risks of bias such as distortion linked to accessibility heterogeneity,^25^^,^^26^ which applies naturally in countries where health care provision is associated with higher socio-economic status. Scandinavian countries have relatively homogenous access to health care and have a tradition of maintaining excellent records, but these data sets are smaller and the populations are less ethnically diverse, limiting generalizability and transferability. A key to delivering on the promises implied by the characteristics of the English data set is their quality and successful linkage of the 83 separate parts of the database including 79 screening centre data sets and 4 data sets about cancer outcomes, invitation records, socio-economic background, and mortality.

In 2009, Bray and Parkin published guidance on the practical aspects and techniques for addressing data quality at the cancer registry, considering comparability, validity, timeliness, and completeness.^27^^,^^28^ This framework has been used for the evaluation of the Swedish breast cancer registry^29^ and cancer registries more generally in the United Kingdom,^30^ Iceland,^31^ Finland,^32^ Norway,^13^^,^^33^ Bulgaria,^34^ Ukraine,^35^ and Singapore.^36^ Other studies examining the quality of cancer registry data focused on completeness only,^37–40^ or on completeness and timing.^41^ The UK government has recently laid out a data quality framework based on the Bray and Parkin framework (Gov guidelines, 12/2020),^42^ but this is the first time such a framework has been applied to breast cancer screening data. Data quality assessment for observational health studies has come under the spotlight due to the risk of misclassification, bias, and hence potential irreproducibility observed; for example, with the use of electronic health records, real-world evidence in pragmatic clinical trials, and repositories such as UK Biobank.^43–46^

The first aim of this article is to describe the construction of the ATHENA-M data set by combining 83 existing data sets from different sources. Through comprehensive linkage of individual women’s trajectories, we provide a rich resource for future studies improving the quality and effectiveness of screening programmes. The second aim of this article is to assess the ATHENA-M data set from a data quality perspective to ensure reproducibility of findings. We set up a framework based on 4 common pillars for data quality, along with concrete quality checks tailored to a composite longitudinal data repository for cancer screening.

## Methods

ATHENA-M is a unique composite data set created from repositories of the National Breast Screening Service (NBSS) at 79 separate breast screening centres, the national invitation system Breast Screening Select, Office of National Statistics mortality data via the Public Health England Mortality and Birth Information System (PHE-MBIS), the National Cancer Registration and Analysis Service (NCRAS), and the Index of Multiple Deprivation derived from postcode (IMD)—see Table 1 below for more information.

**Table 1.** Data sources and pre-processing for ATHENA-M.

National Breast Screening Service (NBSS)	In the first decade of the programme, IT support was decided regionally leading to the use of NBSS and 4 other administrative systems operating locally until NBSS became the nationwide standard in 2004-2005 and has remained largely unchanged since. Data were collected between November 2018 and May 2019 from each of the 79 breast screening services using a standalone set of extract programmes written using SAP Crystal Reports and an Open Database Connectivity (ODBC) interface (standard with the NBSS system implementation), saved in text format and sent to Public Health England for collation and cleaning. Three extracts were taken from each centre: details of eligible women invited to screening (NBSS-women), details of eligible routine screening episodes (invitation for screening and all of the associated actions that happen as a result) (NBSS-episode), and clinical details of screening episodes where the woman was recalled for further tests (NBSS-feature).
National Cancer Registration and Analysis Service (NCRAS)	The systematic collection of cancer and tumour disease data in England is managed by NCRAS with over 300 000 cases of all cancers collected annually, including patient details, cancer type, and information on severity and received treatment. Data from health care providers, histopathology and haematology services, radiotherapy departments, screening services, general practitioners, and other services are matched and merged to build a complete picture of the cancer incidence in England and to understand how cancer patients are diagnosed, treated, and what their outcomes are. Once all expected records for any one incidence year have been received, validated, and quality assured, NCRAS takes a snapshot of the data set providing a single, consistent source of cancer registrations. We used the August 2021 snapshot for linking screening data to registered patients.
Breast Screening Select (BS Select)	The data set of the national invitation system for the NHS breast screening programme in England (BS Select) dates back to the beginning of the programme in 1988 and contains women registered with a general practitioner in England. It is used to automatically send a list of women who are due an invitation, based on the parameters set when creating a batch, to the responsible screening office who imports this list into NBSS. It receives in turn a screen outcome for each of these episodes. NBSS includes all routine screening call and recall appointments, as per study inclusion criteria, but initial data cleaning suggested missing data at a subset of centres. BS Select was used to check whether women eligible for routine call and recall were recorded as other appointment types: self-referrals, general practitioner referrals, higher risk referrals, and non-routine early recall appointments.
Index of Multiple Deprivation (IMD)	As a frequently used compositive measure for relative deprivation in small areas, the IMD captures components such as income, employment, education, health, crime, housing and services, and living environment. In England, it is revised every few years by the UK Ministry of Housing, Communities and Local Government (MHCLG).^54^ ATHENA-M includes the quintiles of the income domain using the women’s postcode at the time of her last screening appointment. To reflect revisions, both a score based on the IMD current (at that time) and on IMD 2015 are included.
Office for National Statistics (ONS) Death Records	The Public Health England Mortality and Birth Information System (PHE-MBIS) was created to streamline the sharing, storage, and dissemination of ONS birth and death registration by PHE. The data were released under the control of the PHE Office for Data Release. Recording of death data on PHE-MBIS started in 1997 (month unknown).

### Population: inclusion criteria

In ATHENA-M, we included all women invited to at least one breast screening appointment in England between ages 47 and 73 years, between January 1, 1988 and March 31, 2018, based on date of first offered appointment. We excluded women without a screening invitation accompanied by date information, and women whose appointment was not part of the standard breast screening programme (for example women who self-referred with symptoms). Women who opted out of having their data being held on the National Disease Registration Service (NDRS) registers had already been applied to the cancer registry data. In line with the National Data Opt-Out policy, this opt out was not applied to the screening data as no confidential patient information was shared with any organization external to Public Health England (PHE) (details in supplementary table C2; the rate of national data opt out at that time was 5.3%).

### Population: screening protocol in England from its initial roll-out until today

**Figure 1.**
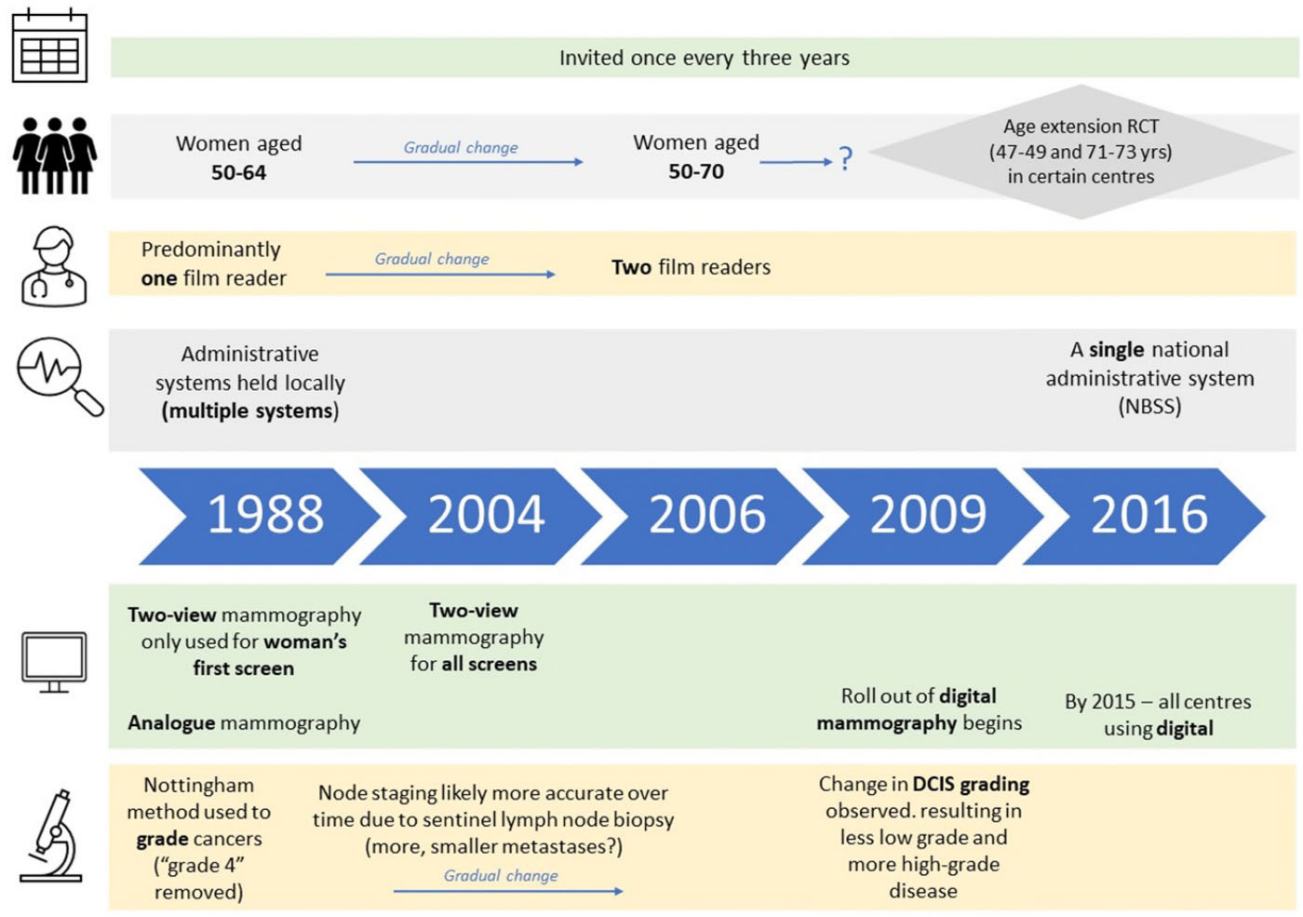
Timeline for changes to policy and process in the English cancer screening programme.

The roll-out of the national breast screening programme for women aged 50-64 years began in 1988 in selected areas and was extended to cover the whole of England from 1990. Each woman is invited once every 3 years. Changes in the programme’s operation include extensions of ages eligible for screening, the increased involvement of a second reader to search for signs of cancer on the mammograms, harmonization of the administrative systems, technological changes, and some modifications to breast cancer classification.^47^ Extensions of the invited population may affect the prevalence of cancer, and improvements in medical diagnostics may affect detection and observed characteristics of cancers. For example, technological developments such as the roll out of digital mammography has increased the rate of Ductal carcinoma in situ (DCIS) diagnosis^48^ which may also have played a role in the change in reported DCIS grade classification (less low, more high grade) that has been observed in parallel,^49–51^ and more accurate node staging may lead to the detection of more metastases of smaller size. Similarly, the evolution of audit and quality assurance processes, and key performance indicators have driven changes in practice.^52^^,^^53^ The modifications most notable for this study are visualized in Figure 1 and described in detail in the supplementary materials.

### Data source/s and pre-processing

The ATHENA-M data set draws on several *a priori* independent data repositories with their own history that need to be characterized and pre-processed. Table 1 summarizes these data sources with more details in supplementary table B1.

### Data linkage

**Figure 2.**
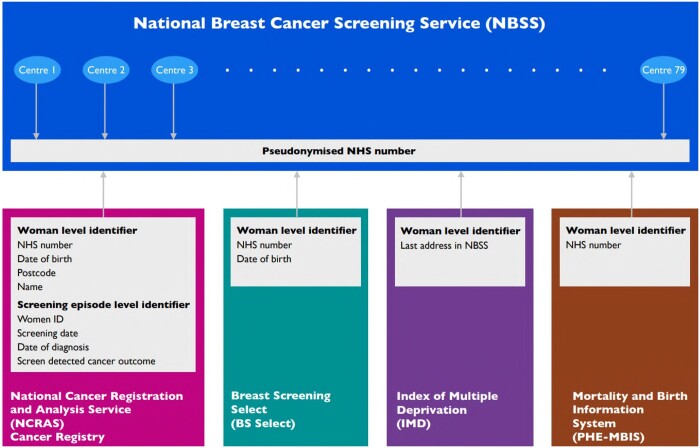
Data sources for ATHENA-M with identifiers used in each linkage shown in grey boxes linking at woman level to the NBSS database via (pseudonymized) NHS numbers allowing matching of a woman’s appointments across different centres.

Figure 2 shows the variables used for linkage between data sets. Linkage between data sets was primarily based on (pseudonymized) NHS number, a unique identifier used across all NHS services for each woman which became universal in 1997 (Figure 5).

**Figure 3.**
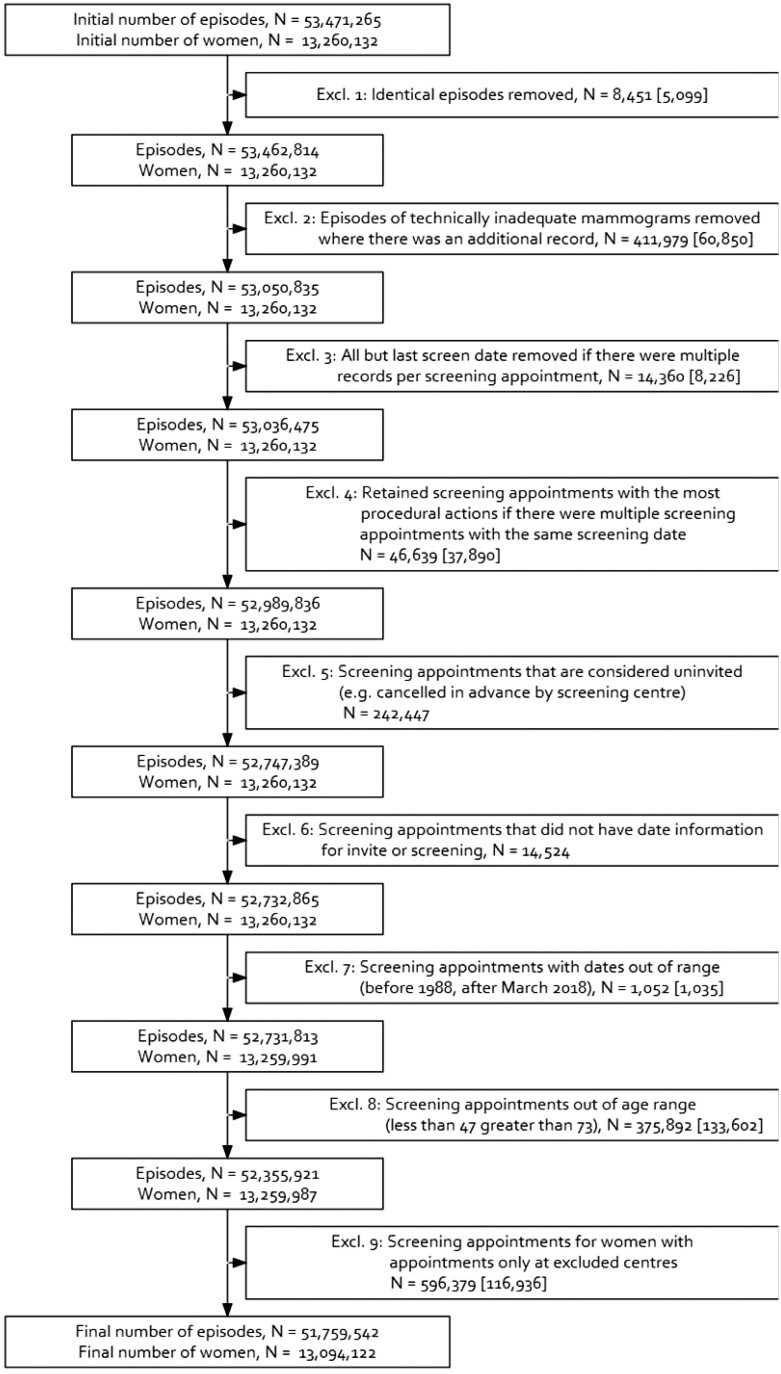
Screening data set exclusion flowchart carried out on NBSS-records with effects on NBSS-women for the whole study period with pre-1997 numbers in brackets (included in the total).

**Figure 4.**
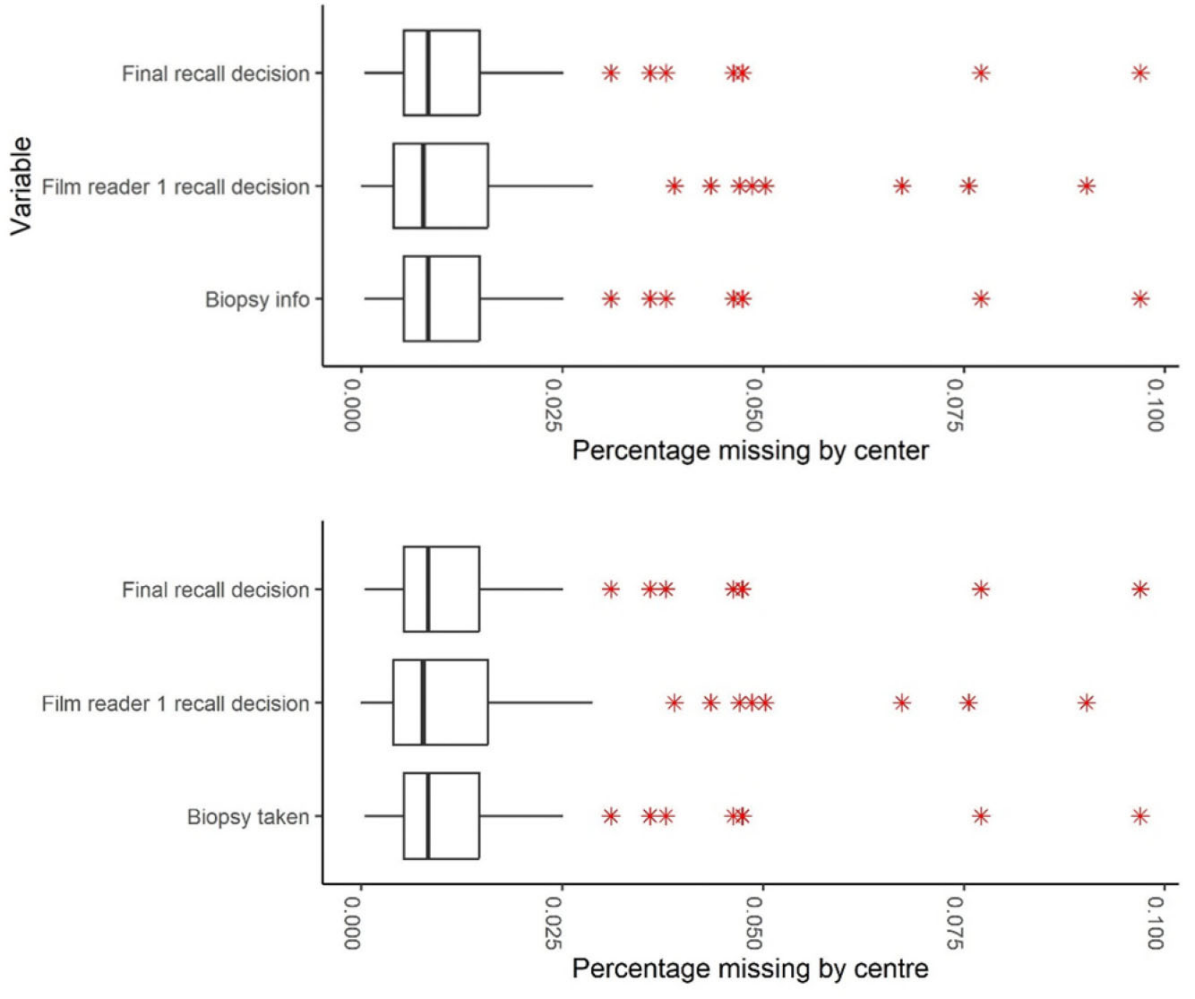
Percentage of missing values in 3 variables by centre post-1997 (excluding 2 centres with <1000 screening episodes).

**Figure 5.**
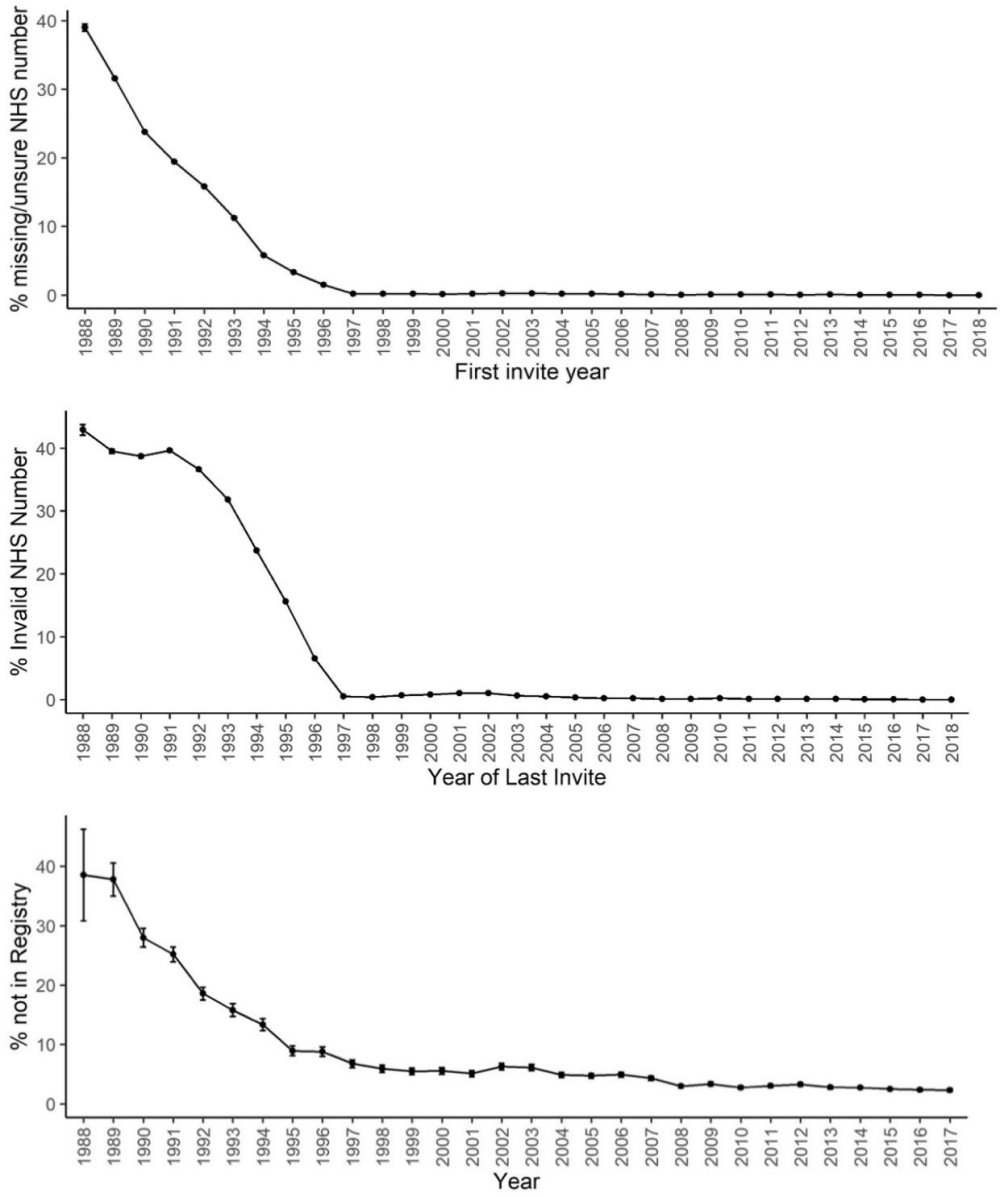
Percentage of women in screening data set without universal identifier (an NHS number in the screening data set or derived by high-quality tracing) by last year of invite over the whole study period (top) and percentage of cancers detected at screening not found in the cancer registry with error bars computed for records missing at random as reference (bottom).

**Figure 6.**
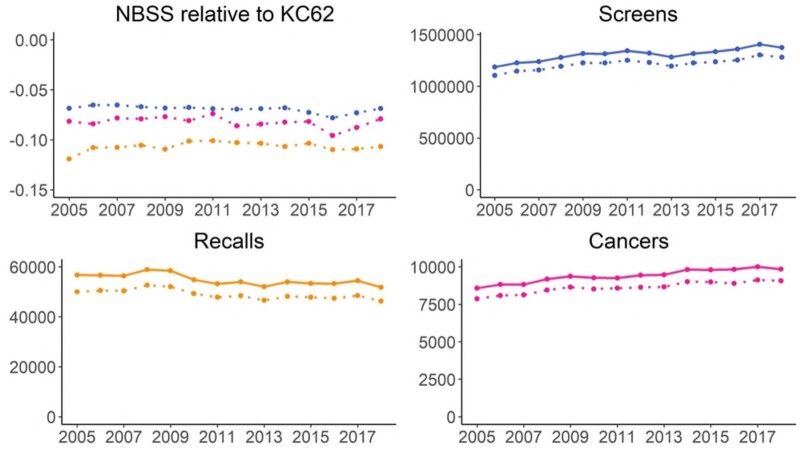
Proportional difference between NBSS data in ATHENA-M data set and KC62 annual return data made by centres each year (ie, difference of NBSS and KC62 counts divided by KC62 counts) for years 2005-2017 (top left graph; top line: screens, middle line: cancers; bottom line: recalls) and comparison of counts of screening appointments, recalls, and detected cancers with NBSS in dotted lines and KC62 in solid lines (top right and bottom row).

**Figure 7.**
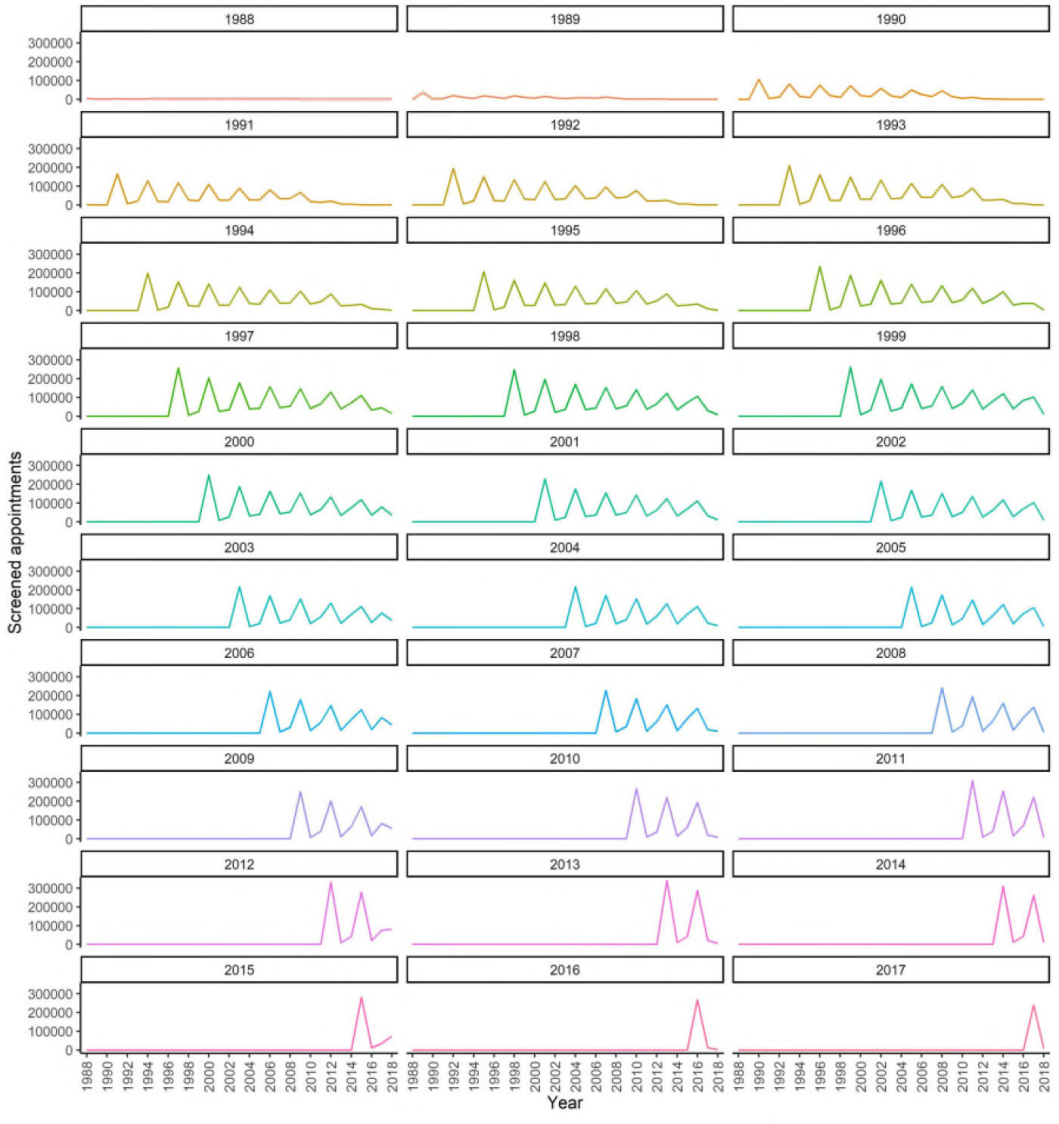
Number of screening invitations by year stratified by first year women were invited.

Records belonging to the same woman using different centres could be matched, but records of the same film reader operating across centres could not be linked. Details about scoring systems used where necessary and record matching counts are shown in supplementary tables B3.2-5. Whilst linkage between the cancer registry and NBSS for each woman could utilize NHS number, there was no identifier linking cancer records for a woman to screening records, and at the point of data extraction the cancer registry did not contain reliable data about whether a cancer was screen detected. NBSS episodes with screen-detected cancer will have the data from the Cancer Registry linked to it if diagnosis date in the Cancer Registry was between 7 days before and 100 days after the “screening date” or “date taken.” The remaining 559 443 cancers in the Cancer Registry are classified as non-screen-detected cancer.

### Quality assessment of ATHENA-M data set

Central pillars for data quality in cancer registries are comparability, validity, timeliness, and completeness.^27^^,^^28^ A rich collection of generic data quality indicators related to these concepts has been suggested to cover a wide range of observational health studies^55^ expanding existing frameworks developed for electronic health records. We adopt these frameworks to assess the quality of composite data about cancer outcomes and the screening journey preceding it. Table 2 shows data quality pillars tailored to the architecture of the ATHENA-M data set and lists the main criteria we used to assess them. We give particular attention to the technological and procedural changes to the screening programme, modifications of administrative processes, changes in cancer classification, and heterogeneity of the invited population. The distinction into crude and qualified missingness addressing nonresponse, drop-out, and other specific reasons^55^ is particularly suited to addressing the missingness occurring through attrition and failed linkage.

**Table 2.** Pillars and criteria to assess data quality in the context of ATHENA-M.

Completeness	Refers to the extent to which screening records, cancer outcomes, and sociodemographic information are included in the database. Missingness can relate to the lack of inclusion of potentially relevant variables or to the lack of values in included variables. In the longitudinal context of screening journeys, missingness needs to be considered across the whole time period.	Missingness in cancer registry variables (Appendix Table SC1) Excluded centres (Appendix Tables SC2.1-3) Missingness of age over the whole study period (%) Missingness in variables from NBSS pre-1997 and later (Table 3 and Figure 4) Failed linkage to cancer registry pre-1997 and later (Table 3) Missing NHS number and failed linkage by year (Figure 5 and Appendix Table SB3.1) Missingness in NCRAS variables pre-1997 and later (Table 3) Non-attended invitations (Appendix Table SC4)
Comparability	Assesses the calibration of the generation of statistics from different population groups associated with different centres, regions, socio-economic status, and demographic characteristics. In our longitudinal setting comparability needs to be addressed over time as well. A basic requirement is standardization of definitions and practices concerning classification and coding of screening and cancer outcomes.	Benchmarking of NBSS data against KC62 data for numbers of screens, recalls, and cancers over the course of the study period (Figure 6) Benchmarking of NBSS data against ONS data for cohort age over the course of the study period (median and IQR)
Validity (accuracy, plausibility, correctness)	Refers to the proportion of cases in the screening results and potential subsequent cancer related outcomes for which given characteristics truly have that attribute. It depends on the precision of the diagnostic process and the level of expertise in abstracting, coding, and recording.	Discordance between cancer indicator and recorded action following screen (%) Consistency between screening and mammography date (%) Recalls for screen detected cancers (%)
Timeliness (currency)	Reflects the degree of updating speed in the screening records and, if applicable, cancer-related outcomes.	Release timelines after censoring (numerical) Time course of screening patterns on the cohort level (Figure 7) Attendance at second screening appointment over time (Appendix Table SC5 and Figure SC6)

Data quality assessment is the basis for reproducible results and has a long tradition in cancer registration originally based on the first 3 pillars.^56^ Timeliness was added because in its absence no accurate trends can be estimated. Completeness is the most straight forward to assess superficially, but its potential implications for statistical inference depend on whether the missing values followed any systematic patterns or not. In the latter case, the occurrence of missing values can be tolerated at relatively high levels, but if the occurrence of missing values in one variable is linked to other variables this seriously impact conclusions. There is a well-developed body of literature defining different notions of random versus systematic missingness, reasons for this, detection strategies, and remedies ranging from imputation techniques in the case of missingness at random to model-based approaches involving knowledge about the missingness patterns.^57^ In practice, optimizing data quality can involve trade-offs such as between timely data and the extent to which they are complete and accurate. Table 2 summarizes how the 4 pillars of data quality were assessed giving a conceptual explanation as well as technical criteria used to derive the findings listed in the “Results” section.

Completeness was assessed at 4 levels. Firstly, the number of centres that contributed data, secondly, missing data in the NBSS data set, thirdly, missing linkage and related to that, fourthly, missing data on cancer and mortality information. In a wider understanding of the assessment of completeness, we also include the issue of uptake of screening appointments. While driven by women’s choices rather than by technical or administrative causes, high levels of appointments where the woman chose not to attend could potentially create similar limitations on the usability of the data set as other types of missing data.

Comparability was assessed by benchmarking the NBSS data against 2 other data sources. Firstly, we compared numbers of screens, recalls, and cancers in the NBSS data with the NHS Breast Screening Programme Central Return Data Sets (or KC62 data). As mandatory requirement, screening centres in England annually submit NHS Breast Screening Programme Central Return data sets (KC62)^58^ with information about processes and outcomes. These are used to monitor management, progression towards achieving targets about cancer diagnosis, and numbers of women screened per centre.^59^ Centres check data completeness in NBSS before running standard extractions for KC62, these extracts are in turn checked by regional quality assurance teams and finally by the national data analytical team. To investigate the amount of missingness, we compared the number of screened women between NBSS and KC62 records by centre annually, between April 2004 and March 2018, inclusive. Specifically, to make records comparable the number of screened episodes from the NBSS data set were grouped by financial year (April to March) and screening service at the time when the screening appointment was sent out. Between April 2004 and March 2018, the age range 50-64 years was used. The KC62 includes additional appointment types such as self-referral which were excluded from our analysis, so whilst we may expect consistent systematic differences this comparison enables identification of any major issues in data extraction or transfer. Secondly, we compared age of women in the NBSS data to ONS data for the relevant age range (50-70 years) for the years 2001-2018 for which ONS data were available. As a population-wide screening programme, this should be similar to national statistics in terms of age distribution.

Validity of the data set was assessed in terms of concordance between different measurement methods for whether a cancer was detected and date of detection, and through logical consistency that every screen detected cancer should be preceded by a decision to recall.

Timeliness was assessed by quantifying the time required for ethical and other approvals, and for data extraction and linkage. Time course profiles are used to study patterns in the number of screening episodes on the cohort level. As in the case of completeness, we adopt a slightly wider understanding of data quality by including aspects of timeliness driven by women’s choices. Specifically, we assess overall screening uptake and attendance to second screening appointments.

## Results

### Exclusions

The initial data set contained records for 13 260 132 women with a total of 53 471 265 screening episodes. As part of the data preparation, all or parts of the records of 276 353 of these women were converted from an invalid or old-style NHS number to a valid 10-digit NHS number using the tracing service, but this service did not work for all.^60^ The screening data set (NBSS-episode) was subject to exclusion steps (Figure 3) some of which also affected the NBSS-women data set. The first 4 exclusions related to duplicated entries (*N* = 8 451, 0.02%), technically inadequate mammograms that were subsequently repeated (*N* = 411 979, 0.77%), and other multiple entries per screening appointment (*N* = 60 999, 0.11%). The following 4 exclusions related to appointment dates classified as uninvited (eg, due to cancellation by the centre), missing date information, dates out of range of the study period (*N* = 258 023, 0.48%), and appointments for women outside the standard age range at screening (younger than 47 years or older than 73 years, *N* = 375 892, 0.70%). Three specific centres had data collection issues, described in supplementary materials (part C). Women who only had data from these centres had all their screening appointments removed (*N* = 596 379, 1.12%), but 345 578 screening appointments at these centres were kept in the data set for women who also used other centres, to facilitate more complete screening records for these women. Supplementary table C2 shows that the 3 excluded centres have very similar characteristics to the other centres which ensures that their removal has a very limited effect on conclusions drawn from this data set. The final data set contained records for 13 094 122 women and 51 759 542 invitations to screening appointments, of which 38 319 093 (74.0%) were attended, resulting in 38 185 530 screens (73.8% of invitations); 2 271 367 (17%) women did not attend any episodes. The initial data set did not contain non-routine appointments as they were not recorded as part of NBSS. However, taking into account also the BS Select records showed that the vast majority (95.1%) of all screening appointments were indeed routine appointment (NBSS records after exclusions). This is followed by self-referrals (3.8%) and GP-referrals (0.7%) as detailed with yearly breakdowns in supplementary table C3.

Between 1st January 1997 and 31st March 2018, a total of 11 262 730 women were offered screening of whom 9 371 973 attended at least one appointment during that time period, with 139 million person-years of follow-up (a median of 12.4 person years for each woman included) with 73 810 breast cancer deaths and 1 111 139 any-cause deaths. In the same time period, there were 238 922 screens with cancer detected and clinically confirmed affecting 236 468 women.

### Completeness (pillar 1)

Completeness in terms of screening centres was affected by the 3 centres that were excluded from the analysis, as discussed in the “Exclusions” section. However, there were no extreme differences between the excluded centres and the included centres regarding screening outcomes (see supplementary table C2).

Age information is nearly complete except for the first few years. While IMD was nearly complete (missingness rates of at most 1.9% in all time periods according to supplementary table C2), ethnicity data are very sparse (only collected by a small number of centres in later phases of the programme).

Data such as biopsy information, film reader recall decision, and final recall decision should be present for every screening appointment. The percentage of records with those variables missing is listed in the upper part of Table 3. Of the 38 185 530 screening appointments, the decision about recall for further tests by the first reader was missing for only 7218 (0.02%) and there was not a valid reader identifier for 676 785 (1.77%). The second reader’s decision is missing for 7 336 330 (19.21%) appointments, but this reflects the gradual introduction of second readers. The final recall decision is only missing for 129 843 appointments (0.34%). Figure 4 shows missingness of these variables post-1997 at centre level for all sufficiently large centres. Missingness of reader and final recall decisions and biopsy information in these centres is generally below 1% and apart from a few outliers even under 0.05%.

**Table 3.** Missingness in screening process, linkage, and cancer characteristics.

	Overall	1997 and later	Pre-1997
**Successful screening episodes**	38 185 530	31 963 548	6 221 982
**Reader 1 recall decision**			
Other^a^	58 934 (0.15%)	33 356 (0.10%)	25 578 (0.41%)
Missing	7218 (0.02%)	4655 (0.02%)	2563 (0.04%)
**Reader 2 recall decision**			
Other^a^	34 146 (0.09%)	22 825 (0.07%)	11 321 (0.18%)
Missing^b^	7 336 330 (19.2%)	3 584 826 (11.2%)	3 751 504 (60.3%)
**Final recall decision**			
Invalid code/missing	129 843 (0.34%)	79 175 (0.25%)	50 668 (0.81%)
**Needle biopsy follow-up tests**			
Missing	6941 (0.02%)	4059 (0.01%)	2882 (0.05%)
**Cancer detected at screening**	271 380 (0.71%)	238 922 (0.75%)	32 458 (0.52%)
Not linked to registry	14 614 (5.4%)	9000 (3.8%)	5614 (17.3%)
Linked to registry	256 766 (94.6%)	229 922 (96.2%)	26 844 (82.7%)
**DCIS**	49 208	45 266	3942
Grade missing/invalid	19 112 (38.8%)	15 461 (34.2%)	3651 (92.6%)
Size missing	32 986 (67.0%)	30 105 (66.5%)	2881 (73.1%)
**Invasive**	207 558	184 656	22 902
Grade other/missing	17 401 (8.4%)	8144 (4.4%)	9257 (40.4%)
Size missing	37 026 (17.8%)	26 612 (14.4%)	10 414 (45.5%)
Node info missing	74 039 (35.7%)	55 791 (30.2%)	18 248 (79.7%)
ER status missing	119 867 (57.8%)	97 215 (52.6%)	22 652 (98.9%)
PR status missing	165 008 (79.5%)	142 183 (77.0%)	22 825 (99.7%)
HER2 status missing	122 828 (59.2%)	100 026 (54.2%)	22 802 (99.6%)
Numerical stage missing	69 309 (33.4%)	51 781 (28.0%)	17 528 (76.5%)
T stage missing	75 218 (36.2%)	58 354 (31.6%)	16 864 (73.6%)
N stage missing	74 222 (35.8%)	56 852 (30.8%)	17 370 (75.8%)
M stage missing	143 229 (69.0%)	122 906 (66.6%)	20 323 (88.7%)

a Other refers to reader decisions that cannot be classified as recall or no recall, including technically inadequate mammograms, and recall with a shorter screening interval.

b A missing decision for reader 2 is often not missing data but represents a screening pathway where there is only one reader examining each woman’s mammograms.

Abbreviations: ER=oestrogen receptor, PR=progesterone receptor, HER2=human epidermal growth factor receptor 2.

A major driver of data completeness was linkage accuracy. In 1988, an NHS number used for record linkage, was only available for 7438/13 019 (57.1%) of women, even after using the tracing service, however, by 1997, missingness was low with 208 240/209 319 (99.5%) of women having an NHS number available (Figure 5 top). The screenings in which a cancer was detected which could not be linked to cancer registry records and for whom therefore data items on the characteristics of the cancer were missing was 59/153 (38.6%) in 1988. After 1996 with mandated use of NHS number this was 9000/238 922 (3.8%) (Figure 5 bottom). As a direct consequence of missing linkage, cancer type (DCIS or invasive) was missing for 14 613 (5.4%) records overall (Table 3).

For further cancer characteristics, missingness was a product of both invalid data linkage and missing data in the cancer registry itself which was substantial before 1997 (Figure 5). For instance, before 1997 information on grade was missing for 3651/3942 (92.6%) DCIS cases and 9257/22 902 (40.4%) invasive cancers, while in the time period from 1997 onwards this reduced to 15 461/45 266 (34.2%) for DCIS and 8144/184 656 (4.4%) for invasive cancer. Missing information on lesion size reduced from 2881/3942 (73.1%) to 30 105/45 266 (66.5%) for DCIS and from 9257/22 902 (40.4%) to 8144/184 656 (4.4%) for invasive cancers. No data are available to explain the greater missingness for DCIS than invasive cancer data, but we do know invasive cancer characteristics were used for quality assurance and clinical management decisions which may have increased completeness of reporting. Information on node involvement, receptor status, and numerical stage of invasive tumours was rarely reported at all before 1997. Further details about missingness in cancer registry variables are listed in supplementary table C1.

Supplementary table C4 shows the distribution of women in the screening data set by the number of non-attended invitations. The majority of women (53.7%) attended all the appointments they were invited to, while 32.5% did not attend 1 or 2, and only 13.8% did not attend >2.

### Comparability (pillar 2)

The use of NBSS records created under the umbrella of the national health system has led to a high degree of consistency in procedures, variables names, and their meaning. It is matched by a similar level of standardization of codes used at the cancer registry. Details can be found in the corresponding sections in the supplementary materials. Successful linkage of these repositories led to an unparalleled level of comparability of the data across the whole geographical area covered by the screening programme.

Benchmarking of the extracted NBSS data against KC62 data showed that the latter had overall slightly higher numbers of screens, recalls, and cancers (Figure 6). The difference can mostly be explained by women who self-refer or are referred by their GP for screening, rather than as part of the standard call-recall system, as these women are included in the KC62 data but not in the screening data. The KC62 data recorded an average of 90 663 more screens per year than the NBSS data, 5857 more recalls, and 773 more women with cancer. KC62 numbers of screens, recalls, and cancers exceeded the NBSS count by at most 7.8%, 11.9%, and 9.6%. The systematic difference was consistent over time and aligns with the expected difference as the ATHENA-M data set excludes self-referral appointment types which are included in KC62.

Comparison of women in the NBSS data with those in the ONS data for the years 2001-2018 shows that from 2006 onwards, the median woman’s age for both the ONS data and the screening cohort from NBSS was 59 years (IQR 54-64). Prior to 2006, women in the NBSS were slightly younger by a maximum of 2 years (2001 median 56 years [IQR 53-60] vs 58 years [IQR 54-64]).

### Validity (pillar 3)

The cancer indicator from NBSS (whether cancer was detected following a screen) was discordant to the recorded action taken following screening from NBSS in 641/38 185 530 (0.002%) of cases. The date of screening and the date of mammography were identical in 38 164 605/38 169 905 (99.986%) of cases. Of the 271 380 screening appointments where cancer was detected, 1602 (0.59%) did not appeared to have decided to recall the woman for the tests required to detect cancer. This is logically inconsistent, but in practice may occur rarely when a woman attends screening and symptomatic service in the same time period, or when unusual pathways are followed after a technical recall.

Generally, validity concerns were low and improved over time for all indicators assessed.

### Timeliness (pillar 4)

The ATHENA-M data set was censored in 2018 and released to researchers in 2022. The process of receiving NHS ethical approval took 4 months, the process of data extraction from all 79 breast screening centres took 9 months, the process of data linkage to other data sets took 11 months and approvals for data release took a further 2 years, partly impacted by the COVID-19 pandemic and reorganization of healthcare structures. These delays provide some of the limitations to timeliness. Further timeliness is challenging to achieve if long-term follow-up to outcomes is required for cohorts receiving 20 years of screening, in the context of changing tests and treatments.

Patterns of screening episodes for cohorts of women by year of first invitation are shown in Figure 7. The 2 first cohorts show no visible patterns due to their small size. A complete screening history of all 7 screening invitations is only available in those initially screened in 1998 and earlier as it covers a timespan of >20 years. There have been significant changes in screening technology, cancer prevalence, and treatment effectiveness since then limiting generalizability of results to modern screening. Patterns show triannual cycles with some delays and decline in participation over the years. The fraction of women who attended second screening appointments within the expected time frame was initially <60%, but quickly rose in the 1990s to plateau around 78%-80% in the decade following 1997 after which is slightly increased and stayed at levels 80%-82% (Supplementary table C5 and figure C6).

## Discussion and conclusions

The creation of the ATHENA-M data set involved 3 phases: acquisition and pre-processing of 5 raw data sources; linkage based on pseudonymized NHS numbers and scoring systems; and exclusions of a very small number of centres and of redundant or erratic individual episode records in other centres. The overall data quality of ATHENA-M is very good. Completeness in the screening journey core variables such as attendance and reader decisions is excellent. Age and IMD score are also nearly complete. Cancer type (DCIS or invasive) is missing in about 5% of cases, but further cancer details have very high missingness before 1997 with moderate improvements afterwards. Ethnicity has only been collected sporadically in a small number of centres. The relatively high level of technical completeness of the data is to a large extent a reflection of the high level of women’s participation in the screening programme (as evidenced by over 86.2% of women attending all except up to 2 of the screening appointments offered to them). It is worth noting that there will also be unknown missing data, for example if a woman emigrated from England to another country, we would have no records; however, we expect these numbers to be very small.

Comparing ATHENA-M to mandatory KC62 records from 2004 onwards shows consistently smaller numbers in screens, recalls, and cancers, but the difference is consistent over time and explainable as missing non-routine cases. Using ONS data as a benchmark, there are small differences in age in the early 2000s, but no noticeable differences from 2006 onwards. Several rounds of exclusions and data cleaning have ensured that records are valid and unique. Validity for screening process variables was confirmed by very low discordance between findings at the screening appointment, resulting actions, and relevant dates. Reader information has a small percentage of invalid values until 2005 but is nearly perfect afterwards which coincides with introduction of automated data entry. Timeliness has been limited by delays in data release processes, and by the nature of the data set where the intervention lasts for up to 20 years. Timeliness in the wider sense as measured by attendance at the second screening appointment within the expected time interval was low initially but quickly rose and plateaued around 1997 (between 78% and 82% in all years since 1997).

ATHENA-M is a large composite data set involving women’s records drawn from 2 levels, centres and screening episodes representative of the English population eligible for breast cancer screening. The data are longitudinal with long follow-up time, especially for the older records, and benefits from using the same NBSS system across the same centres with standardized categorical data collection, large amounts of which are automated. A weakness is the only sporadic inclusion of ethnicity information rendering it unsuitable to address study questions around the role of ethnicity. Another limitation is the high missingness in details about the cancers (grade, size, etc.), especially in the early phases of the study period. This could lead to biased conclusions and confounding.

A US data set with similar aims is presented by Lehman et al.^17^ It used the powerful SEER platform and repositories,^61^ but it only covers 7 years of data in specific geographic areas, which may not be representative of the population of the whole country. They do not have a whole population call-recall system of systematic invitation for all eligible women, so it also may not be generalizable to all women within the geographic area. A Swedish data set of women eligible for screening linked to breast cancers (from the Swedish Cancer Registry) and breast cancer deaths (from the National Cause of Death Register) was established for the evaluation of breast cancer mortality in Swedish breast cancer screening programmes.^62^ One of the strengths of this Swedish screening data set is the high attendance, which is rarely matched, but there are limitations arising from the relative homogeneity of that population.

ATHENA-M is suitable to take on the role of a reference data set for cancer screening evaluation and research. For most objectives, we advise excluding the pre-1997 period when there was no universal unique identifier to ensure complete linkage to outcomes including cancer detection and mortality. Conclusions related to IMD also need to be drawn with care. While the data on IMD is fairly complete, it is based on the woman’s most recent postcode which may not always best reflect the woman’s socioeconomic status (eg, not be up to date, not reflecting where she lived most of her life).

From a data maturity perspective, we identified a set of recommendations for future data collection in this and other population-wide cancer screening contexts:

- Development of a standardized customized data entry format with a user-friendly interface allowing frequent monitoring to ensure and improve data quality;
- Systems of instant data entry by clinical staff in predefined categories presented as user-friendly drop-down lists, without processes requiring clerical staff, and fully automated data collection for those fields where it is possible (such as image metadata, breast density, exposure factors, equipment, compression, reader identifier, reading time);
- Harmonization of definitions related to the screening journey and outcomes to be used across centres, screening records, cancer registry, and in electronic health records in primary and secondary care;
- Introduction of unique reader identifiers to allow linkage of all screens looked at by the same reader within and across centres;
- Use of unique identifiers as suitable surrogates to ensure complete linkage between screening records and cancer registry for both women, and cancer episodes within women
- Improvement of cancer registry data completeness;
- Data linkage to, or collection of, accurate ethnicity information;
- Improvement of data sharing and accessibility to researchers and health care providers, whilst maintaining ethical and data governance standards and ensuring sufficient contextualization to avoid concerns voiced in the context of AI^63^;
- Building in mechanisms to integrate technical innovations, modifications of protocols, or inclusion of additional variables (eg, individual risk factors) in a timely manner.

There are huge potential benefits in data linkage between screening programmes and outcome data, which can be used for research, quality assessment and service improvements, and to underpin data collection for prospective research. In this retrospective study of population-wide English data, we have demonstrated such linkage is possible on a large scale. Using a data quality assessment framework customized to screening journey and outcome data, we found that ATHENA-M has an overall high level of quality. This work can also serve as a guide on how to construct similar data sets for other longitudinal screening programmes.

## Supplementary Material

tqad023_Supplementary_Data

## Data Availability

This project involves data derived from patient-level information collected by the NHS, as part of the care and support of cancer patients. The Cancer Registration data are collated, maintained, and quality assured by the National Disease Registration Service, which is part of NHS England. The Screening data are collated, maintained, and quality assured by the Screening Quality Assurance Service at NHS England. All prospective and retrospective English studies are evaluated by the Breast Screening Research Advisory Committee (RIDAC). Researchers wishing to access data used in this study should contact the corresponding author who will guide them to the contemporary processes.

## Declarations

The RECORD checklist (and extension of the STROBE statement for observational studies using routinely collected health data) has been completed.
