# Supplementary material for: Development and quality appraisal of a new English breast screening linked data set as part of the age, test threshold, and frequency of mammography screening (ATHENA-M) study
Source: Br J Radiol. 2023 Dec 12;97(1153):98–112. doi: 10.1093/bjr/tqad023 (PMC11027252; doi:10.1093/bjr/tqad023)
Supplement: tqad023_Supplementary_Data [file tqad023_supplementary_data.docx]

**Supplementary Materials**

1. **Changes to the Breast Cancer screening programme and cancer classification over time**

**Screening programme**

Between the start of the programme in 1988 and the end of the study period changes to the programme’s operation were introduced.

- The NHS Cancer Plan (Department of Health, September 2000) included the intention to extend screening to cover the age range from 50 to 70. The programme extension was rolled out across the country between 2000 and 2006.
- Initially in most centres two-view (CC and MLO) mammography was only used for prevalent screens with MLO views only in the incident round. Following research in the late 1990s^1^ the recommendation was changed and all screening units in England had extended two-view mammography for incident screens by December 2004.
- When the screening programme began there was mixed practice, with most centres only using a single film reader to examine each woman’s mammograms and decide whether to recall her for further tests. Over time centres moved to two film readers independently examining each woman’s mammograms (called double reading). By December 1996, 76% of units had introduced double reading, and it was standard practice by January 2006. Where the two readers disagreed whether to recall the woman, arbitration by a third reader or group of readers is used. In some centres in some time periods arbitration is used to determine whether to a recall a woman, even if the first two film readers both recommended recall. This is to keep recall rates, and the associated rates of false positive recalls low.
- The Cancer Reform Strategy (Department of Health, Cancer Reform Strategy, December 2007) announced the intention to extend the eligible age range by 6 years to include women aged 47-50^th^ birthday and women in the age group 71-73^rd^ birthday. In 2009 this ‘Age Extension’ was partially rolled out as a randomised controlled trial (RCT) in 65 screening centres. In these centres 50% of women were randomly assigned to receive extra screening. Of the remaining centres, 4 did not participate in the trial, 9 struggled with the randomisation and instead enrolled all, and 2 were closed and their eligible populations were distributed to others. The trial was piloted in 4 centres before the others joined between January 2010 and December 2014.
- Adoption of digital mammography to replace analogue machines was rolled out between 2009 and September 2015.
- The administrative systems receive their population in the form of batches specified from the population register. Pre-July 2016 these were specified on locally held instances of the National Health Application and Infrastructure Services (NHAIS) via Open Exeter. In 2016 Breast Screening Select was developed, which, although populated from the same source, provided a single national system to support the identification of the eligible population. This has enabled the implementation of restrictions and controls to improve the standardisation of cohort identification and national oversight of practice, reducing variability (see Table B1.1).

**Cancer classification**

Breast cancer can be classified according to type, stage, and grade to optimise treatment selection, since degree of malignancy is related to morphological appearance of tumours ^2^.

**Type**: There are two major histopathological types of breast cancer, carcinoma in situ (mainly DCIS) and invasive carcinoma. This distinction has remained constant throughout the study period.

**Stage**: Stage considers size, spread, lymph nodes, metastasis, and the statuses of receptors and development in diagnostics can contribute to improved accuracy. A score between 0 and 4 is use for the size of the tumour, whether the tumour has spread to the lymph nodes, and whether the tumour has metastasized. Staging can also consider oestrogen receptor status, progesterone receptor status and Her2 status. During the study period (from late 1999), node staging has likely become more accurate due to a change from axillary sampling/clearance to sentinel lymph node biopsy (SLNB). There is some retrospective evidence that SLNB picks up more, small metastases compared to axillary clearance (Macaskill 2012) which may have had an impact on staging, though the extent is unknown. There is variation in how SLNB is undertaken resulting in varying sensitivity in finding micrometastases (<0.2mm).

**Grade**: The appearance of the cancer under the microscope considering differentiation of cells and speed of growth is used to as the basis for a number system of 1 (low grade) to 3 (high grade). The grading of invasive cancers has been uniformly reported since the early 1990s using the Nottingham method which itself remained constant^2^. Prior to that the grading system used scores from 1–4, so grade 4 cancers represent old cases. The Nottingham method has been showing moderate agreement, unchanged over time^3-6^. The system of differentiating DCIS into low, intermediate, and high grade is based on the potential for recurrence or progression to invasive cancer following treatment has been unchanged throughout the study period. Grading for DCIS has remained unchanged. However, coinciding with the change from analogue to digital film mammography a change in DCIS grading has been observed resulting in less low grade and more high-grade disease. Studies have reported only fair overall agreement for grade, with modest agreement in the high and low grade categories and poor agreement in the intermediate grade category^7^.

1. **Construction of ATHENA-M**
2. **Data sources – additional information**

**NBSS**

Each centre had its own instance of the system and its own local database. Most widely used was the National Breast Screening System (NBSS) designed in the late 1980s based on a more generic Patient Administrative system known as the ‘Oxford’ system. Other systems were CAMRASS (South-West London, Surrey, West Sussex), Trent (East Midlands), Kodak (Staffordshire), and HSS (East Anglia and Lancashire). While providing similar types of administrative system, data structures differed slightly. Between 2004 and 2005 a new version of the NBSS system was rolled out as nationwide standard and has undergone few substantial changes since. Data from the previous systems were converted accordingly. Data from units that used the old NBSS system were more compatible with the new NBSS structure.

NBSS data was extracted screening centre by screening centre. The three extracts contained the following information:

- **NBSS-women:** Ethnicity (only populated at a subset of centres), month, year of birth, participation in relevant research trials, and issues with data quality of NHS number identifier
- **NBSS-episode:** Screening date, pseudonymised identifiers for the readers examining the mammograms, their decisions, whether the woman was recalled for further tests, and whether cancer was detected
- **NBSS-feature:** recall characteristics such as side of the body and mammographic characteristics such as mass or microcalcifications

**Table B1.1 Systems prior to NBSS**

| **Originally** | **Developers** | **Area covered** | **Became** | **Date converted to NBSS** |
| --- | --- | --- | --- | --- |
| Oxford System | Oxford Regional Computer System | 2/3 of English programmes | Root system for NBSS | N/A |
| Trent | AT&T Istel and then McKesson | Current East Mids region | N/A | 2004/05 |
| CBSS | Healthcare Software Systems | East Anglia & some areas of the North-West | N/A | Data not available |
| CAMRASS | BM Computing | South West London | N/A | Data not available |
| Kodak | Kodak | Mid Staffordshire | N/A | 2001/02 |

**BS Select**

Self-referral is where women request screening themselves, either because they are over the upper age limit to be invited to screening, or because they did not attend their screening appointment and contacted the breast screening centre to re-book more than 6 months after the original appointment date. General Practitioner referral is where the woman’s GP refers her for mammography, either because she has started at a new GP practice and is eligible for screening, or historically symptomatic women were referred via this route. Higher risk screening is for women with a very high risk of developing breast cancer in comparison to the general population. It involves a younger age of initiation and may use tests other than mammography. Some women may later be moved from high-risk screening to the routine triennial call-recall screening. Non-routine early recall appointments are created when the woman is invited for further tests after shorter than the normal recall period.

**Cancer registry**

Details of all breast cancers C50* and DCIS D05* according to the ICD-10 system, or the pre-1995 equivalents of ‘174’ and ‘2330’, respectively, were included, inclusive of both screen-detected and symptomatically detected breast tumours. Data items include ICD classification, morphology, behaviour, grade, size, number of involved nodes, oestrogen, progesterone and HER2 status, Nottingham Prognostic Index, TNM stage, and whether screen detected. Treatment data items include breast surgery (breast conserving, mastectomy) underarm surgery (axillary clearance, sentinel lymph node biopsy), hormone therapy, radiotherapy, and chemotherapy.

**IMD score**

To accommodate revisions, the ATHENA-M dataset includes two alternative IMD-based scores. The first data point is the quintile of the income domain of IMD 2015 based on the woman’s postcode at the time of her last screening appointment. The second data point is based on the same postcode but uses the version of the IMD current at the time of her last screening appointment: Example to illustrate use IMD 2019 if the last screen was 2014 or later, use IMD 2015 if the last screen was between 2010 and 2013, use IMD 2010 if the last screen was between 2007 and 2009, use IMD 2007 if the last screen was between 2003 and 2006, and use IMD 2004 if the last screen was before 2003. A third variable indicates which year’s indices have been applied for each woman.

1. **Inclusion criteria**

**Opting out**

Women who opted out of their data being held on the National Disease Registration Service (NDRS) registers were already removed from the Cancer Registry data, so we have no record of breast cancer in these women. Under regulation 5 of the Health Service (Control of Patient Information) Regulations 2002 (SI 2002/ 1438), data on women who have opted out via the national data opt out does not have to be removed from screening work defined as service improvement by Public Health England. We had intended to remove these women even though it is not a legal requirement but were unable to due to issues with identifying them.

1. **Data linkage**

The major task in the construction of the ATHENA-M dataset was to link NBSS with the other data sources (Figure 2 in main manuscript). Linkage of women between cancer registry and NBSS was based on NHS number, date of birth, postcode, and name, using a scoring system detailed in Tables B3.2-4 below. NBSS episodes with screen detected cancer were linked to cancer registry records if diagnosis date in the Cancer Registry was between 7 days before and 100 days after the screening ‘date taken’. The remaining cancers in the Cancer Registry are considered to be symptomatically detected cancers. Linkage to PHE-MBIS death data was on NHS number alone, after tracing invalid NHS numbers. The vital status of women with invalid non-traceable NHS numbers remains unknown. IMD quintile are based on last address in NBSS, which was also used for linkage. BS Select data is linked through NHS number and date of birth; either matches between any two of the day, month, year parts, or a match on year, with the day and month transposed.

**Missing NHS numbers by year**

The modern 10 digital NHS number was introduced in 1996. Prior to this date services had more latitude in the generation of local numbers. Data were matched through the tracing service to identify NHS numbers for those clients whose screening predated 1996. If the client had a subsequent interaction with the NHS such as an inpatient stay they would have subsequently had a new NHS number in the system and this would have been matched to their record. However, clients who died, moved outside of England, had their record flagged as sensitive or did not have need to access NHS services e.g., all healthcare was accessed via the independent sector would not be traceable.

The table below lists the counts of valid and invalid NHS numbers, year by year, before exclusions. The relative counts of the invalid ones, recorded as proportion missing below, indicates an overall missingness of 4.0% and also shows that the problem concerns primarily the early years of the screening programme before the modern 10-digit NHS number was introduced in 1996. While 42.9% are missing in the first year of the programme, the missingness drops fast, with 523,969 (28.2%) of women between 1988 and 1996, but only 10,187 (0.1%) between 1997 and 2018. In the last decade (2009 to 2018) only 2,352 women (0.025%) (cumulative invalid NHS number column in Table B3.1 in those years) had an invalid NHS number.

**Table B3.1 Missing and invalid NHS numbers by year**

| **Year of last invite** | **Valid NHS Number** | **Invalid NHS Number** | **Total for year** | **Proportion missing** |
| --- | --- | --- | --- | --- |
| 1988 | 7438 | 5581 | 13019 | 42.9% |
| 1989 | 45807 | 29884 | 75691 | 39.5% |
| 1990 | 118129 | 74570 | 192699 | 38.7% |
| 1991 | 154167 | 100985 | 255152 | 39.6% |
| 1992 | 175284 | 101345 | 276629 | 36.6% |
| 1993 | 197024 | 91770 | 288794 | 31.8% |
| 1994 | 213693 | 66299 | 279992 | 23.7% |
| 1995 | 208796 | 38497 | 247293 | 15.6% |
| 1996 | 216505 | 15038 | 231543 | 6.5% |
| 1997 | 208240 | 1079 | 209319 | 0.5% |
| 1998 | 172498 | 650 | 173148 | 0.4% |
| 1999 | 109896 | 726 | 110622 | 0.7% |
| 2000 | 82853 | 687 | 83540 | 0.8% |
| 2001 | 73598 | 764 | 74362 | 1.0% |
| 2002 | 75061 | 763 | 75824 | 1.0% |
| 2003 | 116017 | 690 | 116707 | 0.6% |
| 2004 | 154097 | 718 | 154815 | 0.5% |
| 2005 | 241524 | 686 | 242210 | 0.3% |
| 2006 | 265589 | 522 | 266111 | 0.2% |
| 2007 | 265973 | 410 | 266383 | 0.2% |
| 2008 | 240228 | 140 | 240368 | 0.1% |
| 2009 | 204148 | 248 | 204396 | 0.1% |
| 2010 | 216133 | 348 | 216481 | 0.2% |
| 2011 | 250937 | 247 | 251184 | 0.1% |
| 2012 | 280935 | 204 | 281139 | 0.1% |
| 2013 | 298769 | 405 | 299174 | 0.1% |
| 2014 | 327830 | 316 | 328146 | 0.1% |
| 2015 | 1741661 | 257 | 1741918 | 0.01% |
| 2016 | 2645059 | 327 | 2645386 | 0.01% |
| 2017 | 2711002 | 0 | 2711002 | 0.0% |
| 2018 | 707085 | 0 | 707085 | 0.0% |
| **Total** | **12725976** | **534156** | **13260132** | **4.0%** |

**Data linkage between NBSS and cancer registry: scoring system Percent Invalid NHS Number**

To link NBSS with cancer registry records, each identifier was given a score from 0 (no match) to 3 (exact match) as summarised in the table below. NHS number and date of birth alone would give a score of 6, this is a standard level of matching. Therefore, we included matches of score 6 if NHS number and date of birth matched exactly and matches of score 7 or more regardless of whether NHS number and date of birth were exact matches. Matches of score 5 or lower and score 6 without exact matches for both NHS number and date of birth have been excluded as unreliable.

**Table B3.2 Scoring system for linkage between NBSS and cancer registry**

| **Weighted value** | **3** | **2** | **1** | **0** |
| --- | --- | --- | --- | --- |
| **NHS number** | NHSnumberbest equals CAS NHSnumber and neither are null |  | NHS number exists in one dataset only, indicating a lack of complete data in the other | NHSnumberbest does not equal CAS NHSnumber or both are null |
| **Date of birth** | Exact match | 2 out of 3 date parts match | Year of birth matches, and one dataset has 01/01/yyyy as date, indicating possibly unknown exact date | Any other non-match or null |
| **Postcode** | Complete match, when formatted as 7 characters |  | First 4 characters match | Any other non-match or null |
| **Name** | Exact match of forename and either current or previous surname | Transposition of forename and surname | First 3 characters of forename and first 3 characters of surname match | Any other non-match or null |

**Matching results NBSS and cancer registry**

The table below shows the number of person matches for each combination of scores across the four identifiers, after NHS number tracing. Common reasons for imperfect matches include different spelling of name, same data but in different order (e.g., surname and forename switched), change of name or address (which is plausible because date of screening and date of symptomatic cancer detection may be years or decades apart). Of the women successfully matched, 91% score eight or more.

**Table B3.3 Score combinations occurring in linking NBSS and cancer registry**

| **NHS number** | **Date of birth** | **Postcode** | **Name** | **Score** | **Person count** |
| --- | --- | --- | --- | --- | --- |
| 3 | 3 | 3 | 3 | 12 | 1,327,016 |
| 3 | 2 | 3 | 3 | 11 | 10,728 |
| 3 | 3 | 3 | 2 | 11 | 252 |
| 3 | 3 | 1 | 3 | 10 | 138,399 |
| 3 | 3 | 3 | 1 | 10 | 946,327 |
| 3 | 2 | 1 | 3 | 9 | 1,829 |
| 3 | 2 | 3 | 1 | 9 | 8,765 |
| 3 | 3 | 0 | 3 | 9 | 207,759 |
| 3 | 3 | 1 | 2 | 9 | 31 |
| 3 | 3 | 3 | 0 | 9 | 70,342 |
| 0 | 2 | 3 | 3 | 8 | 91 |
| 3 | 2 | 0 | 3 | 8 | 3,209 |
| 3 | 2 | 3 | 0 | 8 | 771 |
| 3 | 3 | 0 | 2 | 8 | 72 |
| 3 | 3 | 1 | 1 | 8 | 107,949 |
| 3 | 3 | 0 | 1 | 7 | 179,761 |
| 3 | 3 | 1 | 0 | 7 | 31,268 |
| 3 | 3 | 0 | 0 | 6 | 65,098 |

Table B3.4 relates to counts of fully registered breast tumours, diagnosed between 1971 and 2018, for patients linked from the cancer registry to women in NBSS included in ATHENA-M. It gives matches by score and year. Of the breast tumours successfully matched, 94% have a person match score of eight or more.

**Table B3.4 Counts of fully-registered breast tumours**

| **Person match score** | **Tumour count** | **% of total tumour count** |
| --- | --- | --- |
| 12 | 376,272 | 44.93% |
| 11 | 2,856 | 0.34% |
| 10 | 306,226 | 36.57% |
| 9 | 73,416 | 8.77% |
| 8 | 28,061 | 3.35% |
| 7 | 43,438 | 5.19% |
| 6 | 7,200 | 0.86% |

**Table B3.5 Match scores relating to fully registered breast tumours for patients linked from the cancer registry to women in NBSS by year**

| **Year of diagnosis** | **Person match score** | | | | | | | **Total tumours** | **% of Total** |
| --- | --- | --- | --- | --- | --- | --- | --- | --- | --- |
|  | **6** | **7** | **8** | **9** | **10** | **11** | **12** |  |  |
| 1971 |  | 1 | 4 | 5 | 13 | 1 | 21 | **45** | 0.01% |
| 1972 | 2 | 2 | 4 | 7 | 28 |  | 25 | **68** | 0.01% |
| 1973 |  | 6 | 5 | 10 | 20 | 1 | 31 | **73** | 0.01% |
| 1974 |  | 3 | 3 | 7 | 35 |  | 33 | **81** | 0.01% |
| 1975 | 1 | 7 | 4 | 15 | 42 | 1 | 41 | **111** | 0.01% |
| 1976 |  | 8 | 4 | 7 | 52 | 2 | 45 | **118** | 0.01% |
| 1977 | 1 | 10 | 1 | 19 | 56 | 2 | 66 | **155** | 0.02% |
| 1978 | 2 | 9 | 4 | 19 | 58 |  | 64 | **156** | 0.02% |
| 1979 | 6 | 13 | 12 | 13 | 96 | 2 | 88 | **230** | 0.03% |
| 1980 | 2 | 12 | 14 | 29 | 85 | 2 | 104 | **248** | 0.03% |
| 1981 |  | 13 | 11 | 41 | 111 | 2 | 141 | **319** | 0.04% |
| 1982 | 3 | 14 | 12 | 37 | 122 | 4 | 159 | **351** | 0.04% |
| 1983 | 4 | 19 | 17 | 38 | 160 | 2 | 186 | **426** | 0.05% |
| 1984 | 2 | 21 | 17 | 53 | 173 | 1 | 177 | **444** | 0.05% |
| 1985 | 5 | 20 | 14 | 66 | 228 | 4 | 232 | **569** | 0.07% |
| 1986 | 3 | 24 | 22 | 72 | 213 | 7 | 257 | **598** | 0.07% |
| 1987 | 4 | 26 | 32 | 59 | 268 | 5 | 291 | **685** | 0.08% |
| 1988 | 141 | 522 | 359 | 775 | 1,979 | 41 | 1,772 | **5,589** | 0.67% |
| 1989 | 147 | 680 | 418 | 1,028 | 2,437 | 67 | 2,307 | **7,084** | 0.85% |
| 1990 | 177 | 801 | 507 | 1,188 | 3,069 | 69 | 3,245 | **9,056** | 1.08% |
| 1991 | 200 | 875 | 591 | 1,506 | 3,978 | 96 | 4,071 | **11,317** | 1.35% |
| 1992 | 208 | 1,008 | 737 | 1,648 | 4,552 | 92 | 4,612 | **12,857** | 1.54% |
| 1993 | 218 | 1,048 | 675 | 1,703 | 4,771 | 87 | 4,854 | **13,356** | 1.59% |
| 1994 | 241 | 1,126 | 847 | 1,718 | 5,152 | 77 | 5,373 | **14,534** | 1.74% |
| 1995 | 264 | 1,136 | 832 | 1,931 | 5,425 | 115 | 5,755 | **15,458** | 1.85% |
| 1996 | 292 | 1,288 | 877 | 2,030 | 6,028 | 100 | 6,396 | **17,011** | 2.03% |
| 1997 | 352 | 1,417 | 992 | 2,275 | 6,966 | 129 | 7,356 | **19,487** | 2.33% |
| 1998 | 306 | 1,449 | 998 | 2,251 | 7,836 | 127 | 8,321 | **21,288** | 2.54% |
| 1999 | 290 | 1,537 | 1,102 | 2,297 | 8,561 | 131 | 9,105 | **23,023** | 2.75% |
| 2000 | 292 | 1,607 | 1,189 | 2,289 | 8,897 | 135 | 9,529 | **23,938** | 2.86% |
| 2001 | 293 | 1,655 | 1,158 | 2,366 | 9,372 | 132 | 10,107 | **25,083** | 3.00% |
| 2002 | 289 | 1,599 | 1,163 | 2,450 | 9,896 | 154 | 10,749 | **26,300** | 3.14% |
| 2003 | 298 | 1,687 | 1,149 | 2,609 | 10,849 | 128 | 11,772 | **28,492** | 3.40% |
| 2004 | 276 | 1,795 | 1,218 | 2,621 | 11,113 | 135 | 12,758 | **29,916** | 3.57% |
| 2005 | 281 | 1,830 | 1,187 | 2,746 | 11,504 | 149 | 13,932 | **31,629** | 3.78% |
| 2006 | 295 | 1,769 | 1,116 | 2,780 | 11,710 | 151 | 14,534 | **32,355** | 3.86% |
| 2007 | 266 | 1,665 | 1,082 | 2,951 | 11,699 | 96 | 15,187 | **32,946** | 3.93% |
| 2008 | 246 | 1,688 | 1,078 | 2,990 | 12,035 | 90 | 17,110 | **35,237** | 4.21% |
| 2009 | 241 | 1,651 | 963 | 3,119 | 11,604 | 56 | 17,687 | **35,321** | 4.22% |
| 2010 | 259 | 1,628 | 949 | 3,125 | 12,059 | 66 | 18,342 | **36,428** | 4.35% |
| 2011 | 229 | 1,528 | 855 | 3,271 | 11,787 | 60 | 19,522 | **37,252** | 4.45% |
| 2012 | 191 | 1,698 | 1,130 | 2,989 | 14,741 | 64 | 17,928 | **38,741** | 4.63% |
| 2013 | 200 | 1,684 | 938 | 3,272 | 14,731 | 51 | 20,010 | **40,886** | 4.88% |
| 2014 | 163 | 1,756 | 1,039 | 3,110 | 16,491 | 56 | 19,894 | **42,509** | 5.08% |
| 2015 | 164 | 1,562 | 869 | 2,832 | 16,318 | 41 | 20,035 | **41,821** | 4.99% |
| 2016 | 141 | 1,317 | 750 | 2,631 | 16,067 | 48 | 20,349 | **41,303** | 4.93% |
| 2017 | 106 | 1,198 | 599 | 2,355 | 16,232 | 39 | 20,677 | **41,206** | 4.92% |
| 2018 | 99 | 1,026 | 514 | 2,063 | 16,607 | 38 | 21,022 | **41,369** | 4.94% |
| **Total tumours** | **7,200** | **43,438** | **28,061** | **73,416** | **306,226** | **2,856** | **376,272** | **837,469** |  |

**4. Exclusions**

**Centres**

Three out of 79 centres had to be excluded from the analysis since examination of patterns of screening attendance highlighted systematic issues with data extraction.

Centre A: There were technical issues with running the crystal report allowing only the extraction of NBSS-episode data; it was only possible to extract data for the first 30,000 women screened at that centre.

Centre B: There were large numbers (75%) of women in the Table NBSS-episode not linked to a woman’s records within that centre in Table NBSS-women. 64% could not be linked to any centre in Table NBSS-women.

Centre C: 35% of women did not have any associated screening episode.

1. **Data quality pillars**

**Table C1: Number and percent of missing variables from the cancer registry by year group highlighting variables with high missingness Pre 1997 (yellow) and Overall (red)**

| **Variable** | **Pre 1997** | **1997 and after** | **Overall** |
| --- | --- | --- | --- |
|  |  |  |  |
| Diagnosis date | 0 (0%) | 0 (0%) | 0 (0%) |
| Basis of diagnosis of the tumour | 0 (0%) | 0 (0%) | 0 (0%) |
| Basis of diagnosis of the tumour text | 0 (0%) | 0 (0%) | 0 (0%) |
| Site of neoplasm (4-character ICD-10-O2 code) | 32,579 (31.03%) | 0 (0%) | 32,579 (3.95%) |
| Site of the cancer and text description | 0 (0%) | 0 (0%) | 0 (0%) |
| Site of the cancer and text description test | 0 (0%) | 0 (0%) | 0 (0%) |
| Morphology of cancer, original coding system | 1 (0%) | 0 (0%) | 1 (0%) |
| Morphology of cancer, in the ICD-10-O2 system | 32,579 (31.03%) | 0 (0%) | 32,579 (3.95%) |
| Behaviour of cancer, in the ICD-10-O2 system | 32,579 (31.03%) | 0 (0%) | 32,579 (3.95%) |
| Numeric behaviour code of cancer and description | 1 (0%) | 0 (0%) | 1 (0%) |
| Behaviour code of cancer, text | 1 (0%) | 0 (0%) | 1 (0%) |
| Grade of tumour | 0 (0%) | 0 (0%) | 0 (0%) |
| Size of the largest dimension of tumour | 64,421 (61.37%) | 213,515 (29.66%) | 277,936 (33.7%) |
| Number of nodes excised | 85,569 (81.51%) | 298,275 (41.44%) | 383,844 (46.54%) |
| Number of nodes involved | 90,864 (86.55%) | 333,739 (46.36%) | 424,603 (51.48%) |
| Laterality | 0 (0%) | 0 (0%) | 0 (0%) |
| Multifocal Tumour | 89,607 (85.36%) | 550,818 (76.52%) | 640,425 (77.65%) |
| Oestrogen receptor status of tumour | 96,757 (92.17%) | 315,666 (43.85%) | 412,423 (50%) |
| Oestrogen receptor score of tumour | 104,843 (99.87%) | 610783 (84.85%) | 715,626 (86.76%) |
| Progesterone receptor status of tumour | 104,262 (99.32%) | 527,924 (73.34%) | 632,186 (76.65%) |
| Progesterone receptor score of tumour | 104900 (99.92%) | 670,742 (93.18%) | 775,642 (94.04%) |
| Human Epidermal Growth Factor Receptor 2 (HER2) status of tumour | 97,867 (93.22%) | 372,355 (51.73%) | 470,222 (57.01%) |
| Nottingham Prognostic Index Score | 96,116 (91.56%) | 362,686 (50.38%) | 458,802 (55.63%) |
| T stage flagged by the registry as ‘best’ T stage | 78,821 (75.08%) | 269,037 (37.38%) | 347,858 (42.17%) |
| N stage flagged by the registry as ‘best’ N stage | 80,561 (76.74%) | 291,613 (40.51%) | 372,174 (45.12%) |
| M stage flagged by the registry as ‘best’ M stage | 87,290 (83.15%) | 418,559 (58.15%) | 505,849 (61.33%) |
| Best ‘registry’ stage at diagnosis of the tumour | 25,047 (23.86%) | 159,641 (22.18%) | 184,688 (22.39%) |

**Characteristics of excluded centres**

Three centres were excluded from the study upfront due to a variety of irregularities (see section B4). Table C2 provides a detailed list of screening centre performance comparing each of the excluded centres with the other ones, separately for each year in the study period. For most variables there is no obvious evidence that the excluded centres are systematically different, but the IMD in these centres is unusually high making comparison of socio-economic status with the other centres impossible.

**Table C2.1: Comparison of excluded centres versus other centres for the whole study period: screening related variables**

|  | **Year Group** | **Other centres** | **A** | **B** | **C** |
| --- | --- | --- | --- | --- | --- |
| Number of women screened per year | 1988-1992 | 29,851 | 21,717 | 31,789 | 16 |
|  | 1993-1997 | 56,359 | 29,830 | 19,686 | 42,733 |
|  | 1998-2002 | 62,550 | 23,555 | 12,131 | 58,251 |
|  | 2003-2007 | 82,520 | 26,769 | 10,339 | 56,725 |
|  | 2008-2013 | 99,891 | 25,072 | 3,087 | 99,497 |
|  | 2014-2018 | 119,989 | 20,400 | 912 | 98,076 |
| Percent (rank) of women screened recalled for further tests | 1988-1992 | 6.08 (42) | 7.19 (22) | 8.31 (14) | 6.25 (33) |
|  | 1993-1997 | 4.66 (40) | 3.27 (73) | 5.29 (25) | 3.51 (65) |
|  | 1998-2002 | 4.89 (40) | 4.94 (43) | 4.59 (50) | 3.91 (66) |
|  | 2003-2007 | 4.56 (40.5) | 5.22 (24) | 4.53 (43) | 3.61 (65) |
|  | 2008-2013 | 4.01 (39) | 3.53 (57) | 4.83 (12) | 3.65 (52) |
|  | 2014-2018 | 3.92 (39) | 2.23 (78) | 4.39 (26) | 3.66 (52) |
| Percent (rank) of women screened with a screen detected cancer | 1988-1992 | 0.58 (40) | 0.61 (30) | 0.51 (58) | 0 (80) |
|  | 1993-1997 | 0.49 (42) | 0.52 (19) | 0.7 (2) | 0.56 (15) |
|  | 1998-2002 | 0.58 (42) | 0.83 (2) | 0.63 (22) | 0.57 (53) |
|  | 2003-2007 | 0.77 (41.5) | 0.95 (2) | 1.13 (1) | 0.7 (69) |
|  | 2008-2013 | 0.77 (40) | 0.85 (9) | 1.26 (1) | 0.68 (72) |
|  | 2014-2018 | 0.81 (41) | 0.87 (13) | 1.32 (1) | 0.81 (40) |
| Percent (rank) of women screened with false positive recalls with benign biopsies | 1988-1992 | 0.64 (40) | 0.78 (24) | 0.53 (41) | 0 (80) |
|  | 1993-1997 | 0.65 (41) | 0.38 (60) | 0.7 (31) | 0.73 (29) |
|  | 1998-2002 | 0.89 (41) | 0.94 (37) | 0.8 (53) | 0.85 (46) |
|  | 2003-2007 | 0.97 (40.5) | 0.95 (44) | 1.01 (36) | 0.8 (57) |
|  | 2008-2013 | 0.96 (39) | 0.71 (63) | 1.13 (17) | 0.73 (59) |
|  | 2014-2018 | 0.99 (38) | 0.39 (78) | 0.88 (44) | 0.84 (49) |

**Table C2.2: Comparison of excluded centres versus other centres for the whole study period: age**

|  | **Year Group** | **Other centres** | **A** | **B** | **C** |
| --- | --- | --- | --- | --- | --- |
| AgeCalc | 1988-1992 | 57.0 (53.0, 60.0) | 57.0 (53.0, 61.0) | 56.0 (53.0, 60.0) | 51.0 (49.8, 53.0) |
| Unknown |  | 4,646 | 0 | 0 | 0 |
| AgeCalc | 1993-1997 | 56.0 (52.0, 60.0) | 59.0 (55.0, 62.0) | 59.0 (56.0, 62.0) | 56.0 (52.0, 60.0) |
| Unknown |  | 1,529 | 0 | 0 | 9 |
| AgeCalc | 1998-2002 | 56.0 (52.0, 60.0) | 60.0 (56.0, 63.0) | 61.0 (58.0, 63.0) | 56.0 (52.0, 60.0) |
| Unknown |  | 351 | 0 | 0 | 4 |
| AgeCalc | 2003-2007 | 58.0 (54.0, 62.0) | 63.0 (58.0, 66.0) | 66.0 (62.0, 68.0) | 58.0 (55.0, 62.0) |
| Unknown |  | 241 | 0 | 1 | 2 |
| AgeCalc | 2008-2013 | 59.0 (54.0, 64.0) | 64.0 (59.0, 68.0) | 67.0 (60.0, 70.0) | 59.0 (54.0, 63.0) |
| Unknown |  | 68 | 1 | 0 | 2 |
| AgeCalc | 2014-2018 | 59 (53, 65) | 64 (59, 68) | 65 (63, 70) | 60 (57, 65) |
| Unknown |  | 73 | 0 | 0 | 0 |

**Table C2.3: Comparison of excluded centres versus other centres for the whole study period: IMD**

| **IMDQUINTILE_2015ONLY** | **Year Group** | **Other centres** | **A** | **B** | **C** |
| --- | --- | --- | --- | --- | --- |
| 1 - Least deprived | 1988-1992 | 482,923 (19.9%) | 171 (0.8%) | 2,316 (7.3%) | 0 (0.0%) |
| 2 |  | 515,550 (21.3%) | 248 (1.1%) | 2,439 (7.7%) | 0 (0.0%) |
| 3 |  | 496,899 (20.5%) | 210 (1.0%) | 1,627 (5.1%) | 0 (0.0%) |
| 4 |  | 461,879 (19.1%) | 95 (0.4%) | 810 (2.5%) | 3 (18.8%) |
| 5 - Most deprived |  | 421,093 (17.4%) | 36 (0.2%) | 213 (0.7%) | 0 (0.0%) |
| Missing |  | 45,651 (1.9%) | 20,957 (96.5%) | 24,384 (76.7%) | 13 (81.2%) |
| 1 - Least deprived | 1993-1997 | 1,049,672 (22.0%) | 810 (2.7%) | 2,137 (10.9%) | 1,720 (4.0%) |
| 2 |  | 1,110,939 (23.3%) | 1,120 (3.8%) | 2,150 (10.9%) | 2,647 (6.2%) |
| 3 |  | 999,161 (20.9%) | 923 (3.1%) | 1,432 (7.3%) | 4,518 (10.6%) |
| 4 |  | 858,001 (18.0%) | 444 (1.5%) | 713 (3.6%) | 10,393 (24.3%) |
| 5 - Most deprived |  | 723,968 (15.2%) | 169 (0.6%) | 186 (0.9%) | 12,570 (29.4%) |
| Missing |  | 30,581 (0.6%) | 26,364 (88.4%) | 13,068 (66.4%) | 10,885 (25.5%) |
| 1 - Least deprived | 1998-2002 | 1,274,827 (23.0%) | 2,180 (9.3%) | 1,622 (13.4%) | 2,368 (4.1%) |
| 2 |  | 1,332,074 (24.0%) | 2,696 (11.4%) | 1,600 (13.2%) | 3,349 (5.7%) |
| 3 |  | 1,167,344 (21.0%) | 2,160 (9.2%) | 1,122 (9.2%) | 4,978 (8.5%) |
| 4 |  | 967,728 (17.4%) | 1,157 (4.9%) | 560 (4.6%) | 9,988 (17.1%) |
| 5 - Most deprived |  | 770,292 (13.9%) | 569 (2.4%) | 144 (1.2%) | 10,816 (18.6%) |
| Missing |  | 34,345 (0.6%) | 14,793 (62.8%) | 7,083 (58.4%) | 26,752 (45.9%) |
| 1 - Least deprived | 2003-2007 | 1,623,024 (23.4%) | 4,040 (15.1%) | 1,041 (10.1%) | 1,595 (2.8%) |
| 2 |  | 1,680,148 (24.3%) | 4,534 (16.9%) | 1,035 (10.0%) | 2,312 (4.1%) |
| 3 |  | 1,459,279 (21.1%) | 3,400 (12.7%) | 686 (6.6%) | 3,510 (6.2%) |
| 4 |  | 1,185,997 (17.1%) | 1,837 (6.9%) | 339 (3.3%) | 7,032 (12.4%) |
| 5 - Most deprived |  | 930,447 (13.4%) | 864 (3.2%) | 96 (0.9%) | 7,440 (13.1%) |
| Missing |  | 49,463 (0.7%) | 12,094 (45.2%) | 7,142 (69.1%) | 34,836 (61.4%) |
| 1 - Least deprived | 2008-2013 | 1,934,573 (23.2%) | 4,696 (18.7%) | 419 (13.6%) | 1,664 (1.7%) |
| 2 |  | 2,003,228 (24.0%) | 4,960 (19.8%) | 375 (12.1%) | 2,348 (2.4%) |
| 3 |  | 1,751,587 (21.0%) | 3,986 (15.9%) | 278 (9.0%) | 3,507 (3.5%) |
| 4 |  | 1,439,459 (17.3%) | 2,378 (9.5%) | 142 (4.6%) | 6,090 (6.1%) |
| 5 - Most deprived |  | 1,133,671 (13.6%) | 1,158 (4.6%) | 39 (1.3%) | 6,248 (6.3%) |
| Missing |  | 75,190 (0.9%) | 7,894 (31.5%) | 1,834 (59.4%) | 79,640 (80.0%) |
| 1 - Least deprived | 2014-2018 | 2,327,340 (23.3%) | 5,038 (24.7%) | 130 (14.3%) | 730 (0.7%) |
| 2 |  | 2,353,817 (23.6%) | 4,954 (24.3%) | 127 (13.9%) | 940 (1.0%) |
| 3 |  | 2,069,474 (20.8%) | 4,068 (19.9%) | 98 (10.7%) | 1,366 (1.4%) |
| 4 |  | 1,731,308 (17.4%) | 2,296 (11.3%) | 38 (4.2%) | 2,249 (2.3%) |
| 5 - Most deprived |  | 1,389,365 (13.9%) | 1,090 (5.3%) | 17 (1.9%) | 1,884 (1.9%) |
| Missing |  | 100,807 (1.0%) | 2,954 (14.5%) | 502 (55.0%) | 90,907 (92.7%) |

**Reasons for mammography invitations**

Invitations to mammography can be issued for a variety of reasons. The most frequent one is a routine appointment invitation. In addition, there are self-referrals, non-routine (early recalls), GP referrals, and higher risk referrals. Table C3 quantifies this based on data from BS Select for each year in the study period. The table has been populated from two sources, NBSS for routine appointments and BS Select for the other ones. In some instances, the date of the first screening appointment was not available in BS Select. Then, the screening date of the reported screen was used where available and the date the screen was taken otherwise (they are nearly always the same as the screening date with exceptions including recall and a few even less frequent issues). As the table only records year there are some instances where recording appears in the subsequent year, but as this would maximally shift by a year the overall picture, we can gain from this table will not change.

The total number of screening appointment recorded between NBSS (after exclusions) and BS Select is 54,426,307. The vast majority of these took place as part of the routine programme (95.1%), though there has been some variation over time. It drops from an initial 100% in the early years to just below 95% in 1997 and then further to under 93% in the early 2000s until it picks up again stabilising around 95%. This dynamic is large due to increased self-referrals and GP referrals during some time periods. Overall, self-referrals accounted for 3.8% of the total and GP referrals for 0.7%, while all other referral types were rare.

**Table C3: First Offered Year by episode type by frequency and percent (referral dataset BS Select)**

| **First Offered Year** | **Overall**, N=54,426,307 | **Routine**,  N=51,759,542 | **Self-referral**,  N=2,074,471 | **Non-routine (early) recall**, N=164,272 | **GP referral**,  N=419,625 | **Higher risk,** N=8,397 |
| --- | --- | --- | --- | --- | --- | --- |
| 1988 | 34,233 | 34,224 (100.0%) | 3 (0.0%) | 0 (0.0%) | 6 (0.0%) | 0 (0.0%) |
| 1989 | 252,920 | 252,872 (100.0%) | 15 (0.0%) | 24 (0.0%) | 9 (0.0%) | 0 (0.0%) |
| 1990 | 733,986 | 733,721 (100.0%) | 163 (0.0%) | 71 (0.0%) | 31 (0.0%) | 0 (0.0%) |
| 1991 | 1,112,726 | 1,112,106 (99.9%) | 107 (0.0%) | 398 (0.0%) | 115 (0.0%) | 0 (0.0%) |
| 1992 | 1,220,784 | 1,217,046 (99.7%) | 187 (0.0%) | 1,600 (0.1%) | 1,951 (0.2%) | 0 (0.0%) |
| 1993 | 1,273,217 | 1,270,808 (99.8%) | 632 (0.0%) | 1,577 (0.1%) | 200 (0.0%) | 0 (0.0%) |
| 1994 | 1,240,325 | 1,233,055 (99.4%) | 3,181 (0.3%) | 2,712 (0.2%) | 1,377 (0.1%) | 0 (0.0%) |
| 1995 | 1,272,802 | 1,241,403 (97.5%) | 15,745 (1.2%) | 8,790 (0.7%) | 6,864 (0.5%) | 0 (0.0%) |
| 1996 | 1,358,934 | 1,301,211 (95.8%) | 32,803 (2.4%) | 11,936 (0.9%) | 12,984 (1.0%) | 0 (0.0%) |
| 1997 | 1,422,781 | 1,350,039 (94.9%) | 45,094 (3.2%) | 10,706 (0.8%) | 16,942 (1.2%) | 0 (0.0%) |
| 1998 | 1,469,031 | 1,373,901 (93.5%) | 63,133 (4.3%) | 11,031 (0.8%) | 20,966 (1.4%) | 0 (0.0%) |
| 1999 | 1,576,596 | 1,479,369 (93.8%) | 68,987 (4.4%) | 6,059 (0.4%) | 22,181 (1.4%) | 0 (0.0%) |
| 2000 | 1,593,562 | 1,479,390 (92.8%) | 85,533 (5.4%) | 3,382 (0.2%) | 25,257 (1.6%) | 0 (0.0%) |
| 2001 | 1,598,552 | 1,483,338 (92.8%) | 93,607 (5.9%) | 3,109 (0.2%) | 18,498 (1.2%) | 0 (0.0%) |
| 2002 | 1,632,886 | 1,517,078 (92.9%) | 96,650 (5.9%) | 2,709 (0.2%) | 16,449 (1.0%) | 0 (0.0%) |
| 2003 | 1,726,737 | 1,612,566 (93.4%) | 96,842 (5.6%) | 2,368 (0.1%) | 14,961 (0.9%) | 0 (0.0%) |
| 2004 | 1,824,895 | 1,709,622 (93.7%) | 99,267 (5.4%) | 2,210 (0.1%) | 13,796 (0.8%) | 0 (0.0%) |
| 2005 | 1,976,224 | 1,887,993 (95.5%) | 72,697 (3.7%) | 2,368 (0.1%) | 13,166 (0.7%) | 0 (0.0%) |
| 2006 | 2,082,523 | 1,993,639 (95.7%) | 72,273 (3.5%) | 2,712 (0.1%) | 13,899 (0.7%) | 0 (0.0%) |
| 2007 | 2,166,653 | 2,078,793 (95.9%) | 72,803 (3.4%) | 2,122 (0.1%) | 12,935 (0.6%) | 0 (0.0%) |
| 2008 | 2,235,557 | 2,138,181 (95.6%) | 84,840 (3.8%) | 1,908 (0.1%) | 10,628 (0.5%) | 0 (0.0%) |
| 2009 | 2,283,971 | 2,173,667 (95.2%) | 97,612 (4.3%) | 1,540 (0.1%) | 11,152 (0.5%) | 0 (0.0%) |
| 2010 | 2,362,968 | 2,259,370 (95.6%) | 92,222 (3.9%) | 1,274 (0.1%) | 10,090 (0.4%) | 12 (0.0%) |
| 2011 | 2,466,992 | 2,358,941 (95.6%) | 94,688 (3.8%) | 1,116 (0.0%) | 12,224 (0.5%) | 23 (0.0%) |
| 2012 | 2,575,719 | 2,474,237 (96.1%) | 88,723 (3.4%) | 1,127 (0.0%) | 11,603 (0.5%) | 29 (0.0%) |
| 2013 | 2,648,553 | 2,546,400 (96.1%) | 89,452 (3.4%) | 996 (0.0%) | 11,501 (0.4%) | 204 (0.0%) |
| 2014 | 2,764,728 | 2,623,151 (94.9%) | 128,108 (4.6%) | 990 (0.0%) | 11,650 (0.4%) | 829 (0.0%) |
| 2015 | 2,765,574 | 2,639,635 (95.4%) | 114,591 (4.1%) | 928 (0.0%) | 8,791 (0.3%) | 1,629 (0.1%) |
| 2016 | 2,859,613 | 2,745,495 (96.0%) | 103,614 (3.6%) | 874 (0.0%) | 7,455 (0.3%) | 2,175 (0.1%) |
| 2017 | 2,858,466 | 2,735,886 (95.7%) | 112,017 (3.9%) | 690 (0.0%) | 7,354 (0.3%) | 2,519 (0.1%) |
| 2018 | 732,762 | 702,405 (95.9%) | 27,651 (3.8%) | 169 (0.0%) | 1,840 (0.3%) | 697 (0.1%) |
| Unknown | 301,037 | 0 | 121,231 | 76,776 | 102,750 | 280 |

**Non-attendance at mammography invitations**

Table C4 shows the distribution of women in the screening dataset by the number of non-attended invitations. The majority of women (53.7%) attended all the appointments they were invited to, while 32.5% did not attend one or two, and only 13.8% % did not attend more than two.

**Table C4: Non-attended invitations**

|  | N = 13,094,122 |
| --- | --- |
| **Non-attended invites** | **Number (%) women** |
| 0 | 7,033,660 (53.7%) |
| 1 | 2,897,341 (22.1%) |
| 2 | 1,361,688 (10.4%) |
| 3 | 722,097 (5.5%) |
| 4 | 424,816 (3.2%) |
| 5+ | 654,520 (5.0%) |

Table C5 and Figure C6 show the level of attendance at the second appointment only but follows this over the course of the study period. Attendance within expected timeframe was at 58.3% and 56.1% in the first two years of the programme but steadily increased during the 1990s until it plateaued around 78-80% from 1997 onwards and then slightly increased to just above 81.9% in 2007 to stay just above 80%. Note that the table ends in 2014 as information about the attendance at the second screening appointment for later years was not yet available at the time of the data extraction in 2018. For the same reason the last few years of the columns recording attendance as outside expected timeframe, and none are still subject to change; some of the non-attenders will eventually catch up and would move to attendance outside expected timeframe in future data extractions.

**Table C5: Attendance at second screening over the time of the study period**

|  | 1^st^ screening | 2^nd^ screening | | |
| --- | --- | --- | --- | --- |
| Year | Age Median (IQR) at screening | Attendance within expected timeframe | Attendance outside expected timeframe | No attendance |
| **1988**  N = 24,171 | 57.00 (53.00, 61.00) | 14,212 (58.8%) | 3,100 (12.8%) | 6,859 (28.4%) |
| **1989**  N = 179,587 | 57.00 (53.00, 61.00) | 100,961 (56.2%) | 20,775 (11.6%) | 57,851 (32.2%) |
| **1990**  N = 521,646 | 57.00 (53.00, 61.00) | 331,971 (63.6%) | 42,871 (8.2%) | 146,804 (28.1%) |
| **1991**,  N = 757,627 | 57.00 (53.00, 60.00) | 512,225 (67.6%) | 58,629 (7.7%) | 186,773 (24.7%) |
| **1992**  N = 756,089 | 56.00 (52.00, 60.00) | 524,412 (69.4%) | 54,710 (7.2%) | 176,967 (23.4%) |
| **1993**  N = 511,218 | 54.00 (51.00, 59.00) | 364,541 (71.3%) | 44,402 (8.7%) | 102,275 (20.0%) |
| **1994**  N = 298,302 | 51.00 (50.00, 55.00) | 226,552 (75.9%) | 34,382 (11.5%) | 37,368 (12.5%) |
| **1995**  N = 259,285 | 51.00 (50.00, 52.00) | 204,219 (78.8%) | 33,000 (12.7%) | 22,066 (8.5%) |
| **1996**  N = 283,309 | 51.00 (50.00, 52.00) | 227,810 (80.4%) | 36,258 (12.8%) | 19,241 (6.8%) |
| **1997**  N = 290,295 | 51.00 (50.00, 52.00) | 239,576 (82.5%) | 35,128 (12.1%) | 15,591 (5.4%) |
| **1998**  N = 277,639 | 51.00 (50.00, 51.00) | 229,681 (82.7%) | 33,645 (12.1%) | 14,313 (5.2%) |
| **1999**  N = 287,772 | 51.00 (50.00, 52.00) | 235,151 (81.7%) | 39,500 (13.7%) | 13,121 (4.6%) |
| **2000**  N = 273,364 | 51.00 (50.00, 52.00) | 223,065 (81.6%) | 38,921 (14.2%) | 11,378 (4.2%) |
| **2001**  N = 245,911 | 51.00 (50.00, 51.00) | 199,419 (81.1%) | 35,433 (14.4%) | 11,059 (4.5%) |
| **2002**  N = 232,070 | 51.00 (50.00, 51.00) | 188,994 (81.4%) | 32,324 (13.9%) | 10,752 (4.6%) |
| **2003**  N = 233,152 | 51.00 (50.00, 51.00) | 190,606 (81.8%) | 31,414 (13.5%) | 11,132 (4.8%) |
| **2004**  N = 233,418 | 51.00 (50.00, 51.00) | 191,010 (81.8%) | 29,932 (12.8%) | 12,476 (5.3%) |
| **2005**  N = 231,216 | 51.00 (50.00, 51.00) | 190,386 (82.3%) | 27,894 (12.1%) | 12,936 (5.6%) |
| **2006**  N = 238,605 | 51.00 (50.00, 51.00) | 198,203 (83.1%) | 26,920 (11.3%) | 13,482 (5.7%) |
| **2007**  N = 243,689 | 51.00 (50.00, 51.00) | 207,014 (85.0%) | 23,088 (9.5%) | 13,587 (5.6%) |
| **2008**  N = 256,260 | 51.00 (50.00, 51.00) | 217,215 (84.8%) | 24,302 (9.5%) | 14,743 (5.8%) |
| **2009**  N = 265,164 | 51.00 (50.00, 51.00) | 222,969 (84.1%) | 24,861 (9.4%) | 17,334 (6.5%) |
| **2010**  N = 283,128 | 50.00 (50.00, 51.00) | 237,276 (83.8%) | 24,392 (8.6%) | 21,460 (7.6%) |
| **2011**  N = 324,848 | 50.00 (49.00, 51.00) | 270,355 (83.2%) | 27,350 (8.4%) | 27,143 (8.4%) |
| **2012**  N = 346,664 | 50.00 (49.00, 51.00) | 291,239 (84.0%) | 15,550 (4.5%) | 39,875 (11.5%) |
| **2013**  N = 357,031 | 50.00 (48.00, 51.00) | 299,165 (83.8%) | 3,974 (1.1%) | 53,892 (15.1%) |
| **2014**  N = 324,643 | 50.00 (48.00, 51.00) | 270,762 (83.4%) | 1,362 (0.4%) | 52,519 (16.2%) |


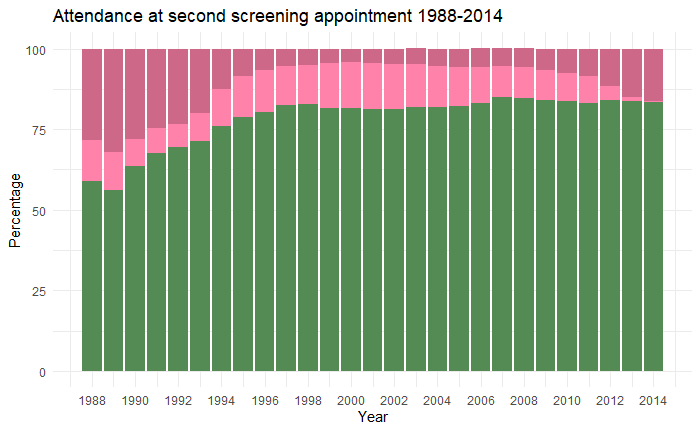


Figure C6. Percentage of women attending their second screening appointment within (green) and outside (light pink) the expected timeframe and not attending it (dark pink) based on available records (relative proportion between outside expected timeframe and no attendance subject to change in later years in future data extraction)

**Appendix references:**

1. Blanks R, Given-Wilson R, Moss S. Efficiency of cancer detection during routine repeat (incident) mammographic screening: two versus one view mammography. *Journal of Medical Screening* 1998;5(3):141-45.

2. Elston CW, Ellis IO. Pathological prognostic factors in breast cancer. I. The value of histological grade in breast cancer: experience from a large study with long‐term follow‐up. *Histopathology* 1991;19(5):403-10.

3. Robbins P, Pinder S, De Klerk N, et al. Histological grading of breast carcinomas: a study of interobserver agreement. *Human pathology* 1995;26(8):873-79.

4. Meyer JS, Alvarez C, Milikowski C, et al. Breast carcinoma malignancy grading by Bloom–Richardson system vs proliferation index: reproducibility of grade and advantages of proliferation index. *Modern Pathology* 2005;18(8):1067-78. doi: 10.1038/modpathol.3800388

5. Ellis IO, Coleman D, Wells C, et al. Impact of a national external quality assessment scheme for breast pathology in the UK. *Journal of Clinical Pathology* 2006;59(2):138-45. doi: 10.1136/jcp.2004.025551

6. Fanshawe TR, Lynch AG, Ellis IO, et al. Assessing agreement between multiple raters with missing rating information, applied to breast cancer tumour grading. *PLoS One* 2008;3(8):e2925.

7. on Breast ECWG, Sloane JP, Amendoeira I, *et al*. Consistency achieved by 23 European pathologists in categorizing ductal carcinoma in situ of the breast using five classifications. *Human pathology* 1998;29(10):1056-62.
